# MUSIFEAST-17: MUsic Stimuli for imagination, familiarity, emotion, and Aesthetic STudies across 17 genres

**DOI:** 10.3758/s13428-025-02724-0

**Published:** 2025-06-20

**Authors:** Hazel A. van der Walle, Wei Wu, Elizabeth H. Margulis, Kelly Jakubowski

**Affiliations:** 1 Department of Music, Durham University, Palace Green, Durham, DH1 3RL UK; 2 Department of Music, Princeton University, Princeton, NJ USA

**Keywords:** Stimulus set, Music genres, Familiarity, Emotional expression, Aesthetic perception, Music-evoked thoughts

## Abstract

Musical stimuli are commonly used in psychological research for investigating a range of emotional, cognitive, and physiological processes. Despite this widespread use, many studies continue to rely on ad hoc music stimulus selection, compromising experimental control, reliability, and comparability across studies. Existing musical stimulus sets tend to be limited in variability in terms of style (e.g. comprising 1–3 genres) and familiarity (e.g. focusing on highly familiar or unfamiliar music). In this paper, we introduce MUSIFEAST-17; a music stimulus set featuring 356 instrumental 30-s clips from commercially released music across 17 genres, aiming to increase the ecological validity of studies tailored for UK and US listeners. Designed to reflect the diversity of everyday Western musical experiences, MUSIFEAST-17 includes art music, popular music, and music composed for media. MUSIFEAST-17 includes normative data from 701 UK and US participants, sampled evenly across adulthood (ages 18–75), on familiarity, enjoyment, emotional expression, perceived contrast, genre recognition, thought types, and contextual associations evoked by each excerpt. Analyses indicated that, while some responses vary by genre, nationality, and age group, MUSIFEAST-17 constitutes a set of stimuli that exhibit stylistic diversity, span familiar to unfamiliar music, and cover a range of emotional expressions. The stimulus set prompted various thought types, including memories and fictional imaginings, and contextual associations such as “movie”, “club”, and “concert”. This resource enables systematic stimuli selection for diverse applications within psychology (e.g. emotion studies, aesthetic experience, music-evoked imaginings) and supports Open Research practices.

**Supplementary Information:**

The online version contains supplementary material available at 10.3758/s13428-025-02724-0.

## Introduction

Music stimuli are increasingly used in psychological research to study emotional, cognitive, and physiological processes. However, many music studies still rely on ad hoc stimulus sets. Considering the number and range of validated sets of other stimulus types, such as words (e.g. De Deyne et al., 2019; Scott et al., 2019) and voices (e.g. Darcy & Fontaine, 2020; Lassalle et al., 2019) that are frequently employed in psychological research, music research is rather behind. Validated stimulus sets are essential for experimental control, reliability, replicability, and comparison across studies. Adopting a more standardised approach to music stimulus selection will allow researchers to explore how participant responses relate to specific stimulus features, enabling a priori predictions and more precise experimental designs, and reducing reliance on post hoc explanations. The availability of online databases and repositories further streamlines access to these resources, promoting Open Research practices, collaboration, and interdisciplinary research endeavours.

Existing music stimulus sets have often been compiled to answer very specific research questions and therefore tend to be limited in scope in terms of the number, stylistic range, and familiarity range of the stimuli. In addition, many music stimulus sets for psychological research comprise simplified stimuli such as single-line (often MIDI) melodies, chord sequences, and otherwise synthesised excerpts (e.g. Baker, 2021; Belfi & Karcirek, 2021; Koelsch et al., 2005; Rainsford et al., 2018). Although this approach adds a level of experimental control, such stimuli are limited in their capacity to evoke strong emotional, imaginative, and aesthetic responses. For research that aims to make broadly generalisable conclusions about the experience of music in everyday life, using’real’, ecologically valid music is essential (Williams & Sachs, 2023).

A key gap in the current provision of music stimulus sets for psychological research is the lack of stimulus sets that cover a wide range of music styles. Several music stimulus sets intentionally restrict stylistic variety, such as the Saraga Carnatic collection dedicated to Indian Art Music (Srinivasamurthy et al., 2021); Yale-Classical Archives Corpus (YCAC) of Western European Classical art music (White & Quinn, 2016); Drum Groove Corpora for studying microtiming (Hosken et al., 2021); Recorded Brahms Corpus (RBC) for performance idiosyncrasies (Llorens, 2021); and Multitrack Contrapuntal Music Archive (MCMA) of Baroque music for contrapuntal and polyphonic independent voices (Aljanaki et al., 2021). However, even beyond these purposefully bounded corpora, music psychology studies often use stimuli with limited genre diversity (e.g. Belfi & Karcirek, 2021; Strauss et al., 2024; Vieillard et al., 2008), and a large proportion of previous research has focused on Western pop music or canonical classical music (Warrenburg, 2020). To capture the richness of everyday musical experiences, and thus the range of psychological responses that accompany these, a broader range of musical genres needs to be considered.

One of the most common topic areas in which standardised music stimulus sets have been employed is emotion research. Several music stimulus sets have been assembled to cover a range of emotions evoked and/or conveyed by music, using either categorical (e.g. happy and sad) (Koelsch et al., 2013; Rainsford et al., 2018; Strauss et al., 2024; Vieillard et al., 2008) or dimensional (e.g. valence and arousal) models of emotion (Aljanaki et al., 2017; Eerola & Vuoskoski, 2011). Some of these also include comparator emotional stimuli in other domains, such as the Ryerson Audio-Visual Database of Emotional Speech and Song (Livingstone & Russo, 2018). Although these stimulus sets cover a range of emotions, they are typically constrained in terms of the range of musical styles and/or familiarity range of the stimuli. One notable attempt to circumvent this limitation is the Previously-Used Musical Stimuli (PUMS) database. PUMS catalogues the range and use of music from over 90 years of music and emotion research, including 22,417 musical stimuli from 306 studies (Warrenburg, 2021). However, while PUMS summarises the stimulus selection of previous studies, it does not include the audio files used in these; as such, its transparency is limited by documentation practices of the original studies. For example, in many instances PUMS is unable to provide details on specific versions or sections of music employed.

Several other musical stimulus sets have been curated to address memory-related research questions. One limitation of existing stimulus sets in this domain is that they tend to include either highly familiar melodies or entirely unfamiliar music, which precludes comparative studies of participant responses to familiar versus unfamiliar music. For example, The Famous Melodies Stimulus Set comprises 107 highly familiar MIDI clips standardised for familiarity based on a US population (Belfi & Karcirek, 2021), and the Corpus d’Extraits Musicaux includes 144 synthesised, monophonic excerpts of tunes expected to be known by “all French-speaking Quebec university students”, standardised for familiarity, age of acquisition, and verbal associations (Peretz et al., 1995). The MUSOS Toolkit comprises 156 novel, intentionally unfamiliar, copyright-free melodies with a computer-based application including modules for recall, recognition, and implicit memory studies (Rainsford et al., 2018) (see also Baker, 2021; Eschrich et al., 2008).

In the related domain of Music Information Retrieval (MIR), there exist many freely available music datasets. This enables different groups of MIR researchers to compare modelling approaches across the same dataset to find optimal solutions to long-standing computational questions, such as automated genre classification (Murthy & Koolagudi, 2018; Tzanetakis & Cook, 2002). MIR datasets often include large numbers of music excerpts across a wider range of genres than typical music stimulus sets developed for psychology research (e.g. the frequently used GTZAN^1^ dataset of 1000 30-s stimuli over ten genres). However, these datasets are not typically normed or curated for psychological research – for example, the metadata included in these datasets often do not contain more information than genre labels and occasional ratings such as valence and arousal (e.g. McKay et al., 2006; Yesiler et al., 2019).

We address these gaps in existing music stimulus sets through the curation of MUSIFEAST-17: MUsic Stimuli for Imagination, Familiarity, Emotion, and Aesthetic STudies across 17 genres. MUSIFEAST-17 includes instrumental clips from ‘real’ (commercially released) music selected based on UK and US listening trends spanning a range of familiarity levels, providing an ecologically valid stimulus set simulating the diversity of UK and US listeners’ everyday musical experiences. Currently, no openly available set of music stimuli for psychological research spans such a wide range of genres and familiarity levels. We offer normative data from across adulthood (ages 18–75 years) from UK and US populations, facilitating age-related stimulus selection and comparison within similar cultures. MUSIFEAST-17’s normative data comprises ratings of clip and style familiarity, enjoyment, and perceived emotional expression (valence and arousal), alongside relatively novel dimensions such as perceived musical contrast, genre recognition, contextual associations, and thought types elicited by the stimuli. These latter dimensions were selected for their prevailing theoretical and practical relevance to research on imagination, memories, and creativity in music cognition, where the nature of internal experiences and contextual interpretations play a central role (Loui et al., 2023; Margulis & Jakubowski, 2024; Thompson et al., 2023). By including ratings on the perception of style and change within the music, the kinds of thoughts it elicits, and the situational contexts it evokes, MUSIFEAST-17 enables a richer investigation of music’s cognitive and emotional impact beyond affective response. With its range of normative ratings across an array of key facets of music, MUSIFEAST-17 offers a versatile and diverse resource for various lines of inquiry within cognitive psychology, empirical aesthetics research, and beyond.

## Method

### Materials/stimuli

#### Music stimulus collection

All music stimuli and datasets generated and analysed during the current study are available in the MUSIFEAST-17 repository, https://osf.io/5ebz2/.

A systematic approach was used to collect music clips across diverse genres and familiarity levels. Seventeen genres, selected based on UK and US listening statistics (Statista, 2024a, 2024b), were targeted for stimuli collection. We aimed to reflect popular listening habits and ensure a broad representation of Western musical styles while excluding less clearly defined categories such as ‘world music’ and ‘religious music’. Genres chosen were: Ambient, Classical, Country, Dance, Electronic, Film music, Folk, Funk, Hip-hop, Jazz, Metal, Pop, Rhythm & Blues (R&B), Rock, and Video game music. We also included two subgenres from specific time periods – Sixties pop and Eighties pop—to facilitate future research comparing responses across different age groups. Our inclusion criteria specified that clips should have sections of at least 30 s with no lyrics, although we allowed for non-lexical vocables (e.g. “oh”, “la”).^2^ This allowed us to focus on the impacts of the music itself, rather than the semantic content of the lyrics, on participant ratings (e.g. emotional valence, contextual association).

A total of 357 music clips were collected, 21 from each genre, with seven clips per genre anticipated to be high, medium, and low in familiarity. However, one Sixties excerpt was excluded post hoc due to an editing error, as the section included in the study contained lyrics; thus, our final stimulus set and all subsequent analyses include 356 clips. To select stimuli based on intended genre and familiarity, we used online platforms IMDb (for Film and Video game music) and Last.fm (for all other genres). With Last.fm, we navigated to the specific genre tag page (e.g. for Metal, https://www.last.fm/tag/metal) and viewed results by “Top artists”.^3^ For high familiarity clips, the artists on the first page of results were examined in order; their “Top tracks” viewed by “All time” were considered until a track was found with an instrumental section of at least 30 s long. If no suitable tracks were found from a particular artist, the next artist in the “Top artists” list was considered. The above process was repeated for low and medium familiarity excerpts. For low familiarity, collection started from the final page of artists and worked backwards, and for medium familiarity, we navigated to page 12 of “Top artists” and proceeded forwards from there.

For the purposes of our study, we required that the artist’s music was on Spotify and that the song belonged to the selected artist (i.e. their name was first credited under the song, not a feature or remix of someone else’s song). For Sixties and Eighties music, the song had to be originally released in 1960–1969 or 1980–1989, respectively. For low familiarity Sixties and Eighties, the extra tag “pop” was added (e.g. https://www.last.fm/tag/60s-pop) to keep these stimuli within the broader “pop” genre (i.e., not merely collecting seven obscure tracks released in that decade). To overcome challenges in sourcing low-familiarity Classical clips (well-known pieces performed by lesser-known artists tended to dominate the last pages of “Top artists” in Last.fm), we used Classical music from a previously validated stimulus set rated as low familiarity (Jakubowski et al., 2024). The criterion that the track must belong to the artist was less relevant to Jazz, given that Jazz standards frequently become popular through covering artists. Jazz stimuli were thus collected slightly differently by including popular and recognisable Jazz standards (Standard Repertoire, 2021) in the high familiarity sets and ensuring that tracks chosen for medium or low familiarity sets weren’t popular Jazz standards being covered by a lesser-known artist.

Film and Video game music were identified via IMDb, by selecting the “Advanced title search” option with title type “Movie” sorted by “US box office” and title type “Video game” sorted by “Number of ratings”, respectively. For high familiarity, “Movie” was sorted “high to low” and “Video game” by “descending”, starting from the top and working down. Maintaining this sort for medium familiarity, the total number of entries (659,777 in “Movie”, 36,974 in “Video game”, as of January 2024) was divided by 200, rounded up to the nearest whole number, and worked down from that entry number (3299 for “Movie”, 185 for “Video games”). For low familiarity, “Movie” was sorted “low to high” and “Video game” by “ascending”, starting from the top and working down. Criteria for inclusion of Film and Video game music were that the original soundtrack album was available on Spotify, the track had to be composed specifically for the respective film or video game (i.e. not licensed songs from other bodies of work), and only one track per series or title would be included (the first appearing title in the list). For example, regarding “Video game”, there were several *The Last of Us*, *God of War*, and *Uncharted* games in the top-rated game series, so music was chosen only from the first appearing title (e.g. music collected from *The Last of Us* and skipping *The Last of Us 2*). The same approach was taken with “Movie” (e.g. several *Avengers* and *Star Wars* movies in the top US box office numbers).

We added a 0.5-s fade in and out to every 30-s music clip using *ffmpeg*. Clip volume was not normalised across the stimulus set or within genres; stimuli were collected from Spotify which has volume limits on uploads so the music would already be set within a safe boundary, and volume dynamics are an important, defining feature of different genres (Kirchberger & Russo, 2016). All clips are available under educational use and fair use for academic research.

#### Main study questions and tasks

Study questions were compiled into surveys made on *Pavlovia* and integrated into the study created in *PsychoPy* (Peirce et al., 2019). After each 30-s music stimulus, participants completed a “Clip response” survey (Appendix A) that included multiple-choice questions on thoughts type(s) experienced while listening to the music clip, genre categorisation (including an option to choose up to two genres), perceived decade of creation of the music, and perceived continent of creation. Likert ratings (on 1–5 scales) were used to collect participant’s perceived contrast (changes in the music throughout the clip), clip familiarity (recognition of the specific clip), style familiarity (familiarity with the style of the music clip), enjoyment of the music, and valence and arousal (perceived emotion rather than felt). A two-dimensional (valence and arousal) model of emotion was used in this study as it has been shown to more reliably capture perceived emotion in music than a discrete or categorical model, particularly for stimuli that may be emotionally ambiguous (Eerola & Vuoskoski, 2011). Finally, a free response question provided participants with an open textbox to describe what context (i.e. setting, situation, place, time, etc.) they could expect to hear the clip in or associate it with.

Additionally, a “Genre exposure” survey asked participants to indicate how frequently they listened to each of the 17 genres before today (1–5 Likert scale from “never” to “regularly”). A “Final questions” survey at the end of the study asked participants about general demographic information and musical background (see Appendix A), including one question from the Ollen Musical Sophistication Index on self-reported musicianship (Ollen, 2006).

## Participants

Participants were recruited and pre-screened via *Prolific* (Prolific, 2023). Pre-screening ensured participants were from the UK or US (“Place of most time spent before turning 18” had to be UK or US) and evenly distributed across four age bands (18–30, 31–45, 46–60, and 61–75). We also balanced gender recruitment evenly and ensured equal representation from both the UK and US.

A total of 701 participants completed the study, ages 18–75 years (*M* = 44.74, *SD* = 15.44; 344 female, 345 male, 10 other, 2 preferred not to say). 344 reported to be currently living in the UK, 356 in the US, and one in the United Arab Emirates. 334 participants (47.6%) reported being born in the UK and 333 (47.5%) in the US. 685 participants (97.7%) spoke English as their native language. Eighty-five participants (15.4%) were multilingual, reporting being able to fluently speak Spanish (3.4%), French (2.7%), and English (2.6%) as their secondary language, among others. 25% of the sample reported having completed A-Level, Scottish Highers, or a US High School Diploma as their highest education qualification, 4.9% were pursuing an undergraduate degree, 42.5% had completed an undergraduate degree, 2.6% were pursuing a graduate degree, and almost 15% had completed a graduate degree. 6% of participants reported not wearing headphones during the study, and 3.3% reported mild to low-level hearing impairments or corrections (e.g. “Tinnitus, no correction”, “Minor age-related hearing loss”, “I wear hearing aids”); none of these participants failed the audio attention check or reported music hearing difficulties, so their data was retained. Finally, 22.7% classified themselves as non-musicians, 53.9% as music-loving non-musicians, 15.3% as amateur musicians, 4.9% as serious amateur musicians, 2.7% as semi-professional musicians, and 0.6% as professional musicians. Participants’ mean prior exposure ratings to each of the 17 genres highlighted Pop, Rock, Eighties, and Film as the most listened to genres, with Metal, Jazz, Folk, and Funk as the least.

## Procedure

The 357 music clips were divided into 21 stimulus groups, each containing 17 clips (one from each genre), with preassigned clip familiarity levels evenly distributed within each group. Participants were randomly assigned to one of these stimulus groups using *Pavlovia’s* online counterbalancing feature to obtain an even distribution of participants from each of the four age groups and two nationalities for each of these stimulus groups. Each stimulus group was heard by 30 to 38 participants in total. Stimulus group clip allocations and participant count per group can be found in our “MUSIFEAST-17 Normative Data Tables” files on OSF.

Participants were first presented with an information sheet and asked to confirm their informed consent to participate in the study. They then encountered a volume check page with two 7-s clips, relatively quiet and loud, with instructions to adjust their device volume to a level at which both clips could be heard comfortably and to maintain that level throughout the study. Participants were instructed to relax, listen alone and in a quiet environment, preferably with headphones, and avoid distractions.

Participants then listened to the 17 music clips from their assigned stimulus group in a random order. After each clip, the screen automatically progressed to present them with questions regarding their responses to the clip they just heard (thought type(s), familiarity, emotional expression, etc.). An attention-check computer-generated spoken word track was presented after the 8th clip (halfway) instructing participants to type “banana” in a textbox presented on a blank screen. After listening to and rating all 17 clips, participants completed the genre exposure rating survey and demographics questions.

## Analysis

The aims of our analyses were to provide an overview of the normative rating data (e.g. familiarity, valence, arousal, etc.) for the music clips, examine the interrelations between these ratings, consider how ratings varied across different age groups and nationalities (US/UK), and present exploratory overviews of the contextual associations and thought types evoked by the music. More comprehensive analyses of thought-type occurrences in relation to clip ratings will be reported in a separate paper.

Of the 701 participants who completed the study, two participants’ responses were partially excluded: one reported they could not hear the 10th clip, and the other did not see all genre options in the genre categorisation question. The affected portions of their data were marked as “NA” for analysis. Further screening of the data showed no response input or collection issues from *Prolific* or *PsychoPy* and attention checks were answered sufficiently so no additional participants were removed. All analyses were conducted in *RStudio* (version 2024.04.1 + 748) (Posit team, 2025). Each clip in MUSIFEAST-17 was rated by 30–38 participants. Strauss et al. (2024) recommend a minimum of ten raters per clip for stable estimates of categorical music-evoked emotion terms. In this present study, the minimum number of raters exceeds these recommendations. Using R’s *pwr* package, a power analysis was run which showed that, at 80% power, our inclusion of 21 music excerpts per genre sufficiently enabled detection of small to medium differences between 17 genres (Cohen’s ω = 0.24; with medium effect size traditionally considered to be ω = 0.30; Cohen, 1988).

To probe whether the stimulus set covers our intended familiarity range, a Kruskal–Wallis H test followed by post hoc pairwise Wilcoxon signed-rank tests (Bonferroni corrected for multiple tests) were used to investigate how participants’ familiarity ratings of the stimuli compared with our preassigned familiarity levels (high/medium/low). To examine demographic variations across the stimulus ratings, we analysed the effects of participant nationality (UK/US), age group (18–30, 31–45, 46–60, 61–75), genre, and their interactions on ratings of clip familiarity, style familiarity, contrast, enjoyment, valence, and arousal (musical background (*r*s = – 0.074 to 0.160) and education level (*r*s =  < 0.001 to 0.062) showed relatively low correlations with all clip ratings and thus were not explored further). Pearson’s correlations were computed between these clip ratings, followed by a MANOVA model including all clip ratings as dependent variables. Significant effects that emerged in the MANOVA were investigated further via ANOVA models using the *aov_car* function from the *afex* package. ANOVA results were corrected using Greenhouse–Geisser (GG) adjustments for violations of sphericity. Post hoc pairwise comparisons using Bonferroni corrected estimated marginal means (EMM) with the *emmeans* package were performed to explore significant differences across the levels of nationality, age group, and genre. Sum-to-zero contrasts were used in post hoc analyses of the effects of genre on the clip ratings.

To determine if certain subsets of music excerpts tended to be rated similarly in their stimulus features, K-means cluster analyses were performed using the *fvis_nbclust* function from the *factoextra* R package. We were particularly interested in exploring whether genre categories systematically vary in their clip ratings and if certain genres fall entirely within specific clusters. The optimal number of clusters was determined using both the “elbow” method based on the total within-cluster sum of square (WSS) and the silhouette method, which compares similarity of items within versus between clusters.

To provide an exploratory overview of participants’ contextual associations for the music from their open-text responses, word clouds were generated using the *textstem*, *tm*, *stopwords*, *tidytext*, *wordcloud*, and *RColorBrewer* packages in R. Common and custom stopwords (see Appendix B) were removed and the text was lemmatised before generating the word clouds. Custom stopwords were added so the word clouds weren’t dominated by musical descriptors such as genre names or other common non-descriptive words such as “made”, “sort”, or “unsure”.

## Results

Here, we present a comprehensive overview of the range of normative ratings collected for the MUSIFEAST-17 stimulus set. Raw data, clip-level summaries, and Supplementary Material for MUSIFEAST-17 can be accessed at: https://osf.io/5ebz2/.

## Perceived geographic location of origin, genre, and creation decade

As one of our aims was to create a music stimulus set representative of the Western listening context, we asked which geographic location participants perceived each clip to be from. Averaged over the 356 clips, 342 clips (96%) were rated most frequently as Western music (252 rated most frequently as originating from USA/Canada, 90 from Europe). Of the remaining 14 clips, two were rated most frequently as originating from Africa (0.6%), six from Asia (1.7%), and six from Latin/South America (1.7%). The most frequently chosen creation locations summed across all trials were USA/Canada (70.8%) and Europe (25.3%).

**Fig. 1:**
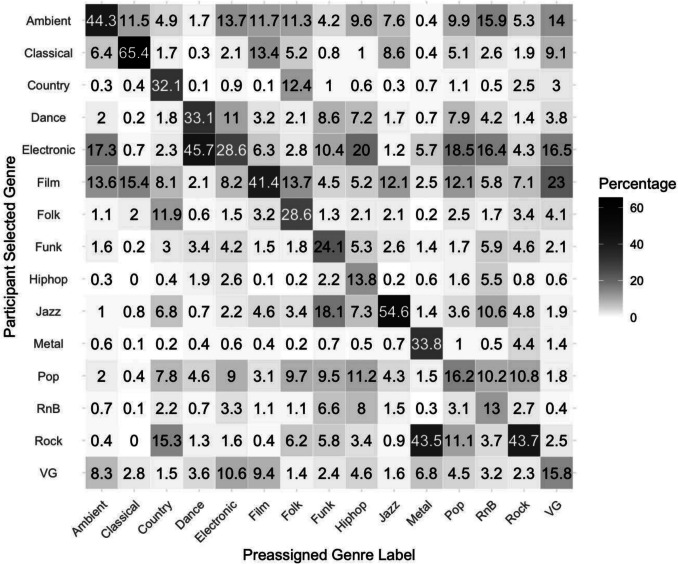
Heat map confusion matrix of between-participant agreement on genre perception, percentage of genre ratings summed over every trial of primary and secondary genre responses (VG = Video game)

A second aim was to create a stimulus set that was perceived as stylistically diverse. To assess whether we achieved this aim, we compared participants’ genre identification of each clip to the preassigned genre labels from Last.fm and IMDb. Overall, participants’ selections matched the preassigned genre label of any given music clip 61.6% of the time.^4^ The genres for which participants’ selections most frequently matched the preassigned genre label were Classical, Jazz, Ambient, and Rock (see Fig. 1). The least frequently matched genres were R&B, Hip-hop, Video game, and Pop. The most frequent mismatches were Dance being labelled by participants as Electronic, Metal as Rock, Video game as Film, and Hip-hop as Electronic.

Examining individual clips, the ten most frequently genre-matched clips were six Classical clips, two Jazz clips, one Country clip, and one Film clip (75.7–91.9% genre-matches over all trials). The ten most frequently genre-mismatched clips were four Metal clips, one Hip-hop clip, one Funk clip, one Video game clip, one Country clip, one Film clip, and one Pop clip (57.1–65.1% mismatches over all trials).

Figure 1 also provides insights into between-participant agreement, as indicated by the magnitude of each selected genre percentage. That is, regardless of the preassigned genre labels, we can examine how often participants agreed with each other about a genre label. Between-participant agreement was highest for Classical (65.4% Classical), Jazz (54.6% Jazz), Dance (45.7% Electronic), and Ambient (44.3% Ambient). Genres with the lowest between-participant agreement were R&B (16.4% Electronic), Pop (18.5% Electronic), Hip-hop (20% Electronic), and Video game (23% Film).

In analogy to our genre perception analyses, we tested whether Sixties and Eighties music clips were perceived by listeners as belonging to their respective creation decades.^5^ Sixties music was correctly rated to be from the 1960s 22.7% of the time, while Eighties music had a higher overall correct identification rate of 48.2%. At the level of individual clips, 14 of the 20 Sixties clips were identified correctly above chance level (0.125), and all 21 Eighties clips were identified correctly above chance level.

## Comparing clip familiarity ratings and preassigned familiarity labels

Another objective for MUSIFEAST-17 was to include clips across a range of familiarity levels. As such, we tested whether participants’ clip familiarity ratings reflected our preassigned familiarity labels (high, medium, and low) across the stimulus set. We anticipated variability within and between genres; certain genres are likely to be more widely known than others, while for some genres, even highly “popular” excerpts are likely to be familiar to a more niche subset of the population.

Over all genres, a significant difference in participants’ clip familiarity ratings was found across the three preassigned familiarity labels (*χ*^*2*^ (2) = 587.42, *p* < 0.001). Pairwise Wilcoxon rank-sum tests further confirmed significant differences between all preassigned familiarity label pairs (High * Low: *p* < 0.001, High * Medium: *p* < 0.001, Medium * Low: *p* < 0.001).

**Fig. 2:**
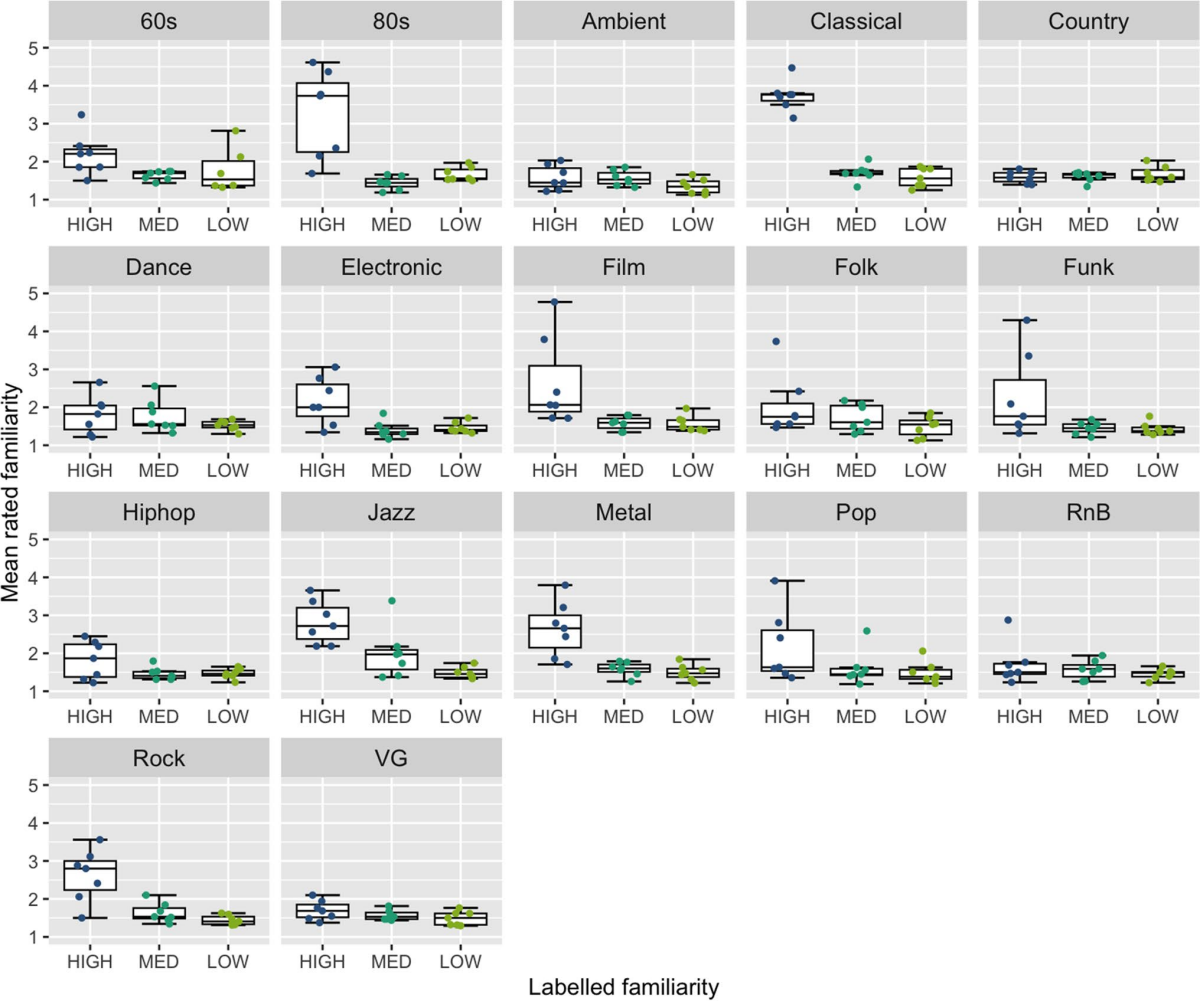
Boxplots of mean clip familiarity ratings (y-axis) across preassigned familiarity labels (x-axis), grouped by music genre. Mean familiarity ratings of each clip are jittered for visibility

For individual genres, Kruskal–Wallis tests indicated that all genres showed significant differences in clip familiarity ratings between the preassigned familiarity labels except Country (*p* = 0.788), R&B (*p* = 0.273), and Video game (*p* = 0.282) genres, which were generally rated as relatively low in familiarity (see Fig. 2). Of the genres with significant Kruskal–Wallis test results, pairwise Wilcoxon rank-sum tests revealed that Eighties, Jazz, and Rock music showed significant differences between all three familiarity label pairings. Unexpectedly, for Eighties music, the preassigned Low familiarity clips were rated significantly higher in clip familiarity than those preassigned as Medium familiarity. Sixties, Classical, Electronic, Film, Funk, Metal, and Pop genres displayed significant differences between only the High * Low and High * Medium familiarity labels; Folk only between High * Low and Medium * Low; Hip-hop only between High * Medium; and Ambient and Dance only between Medium * Low.

## Visualising emotional expression in valence and arousal space

**Fig. 3:**
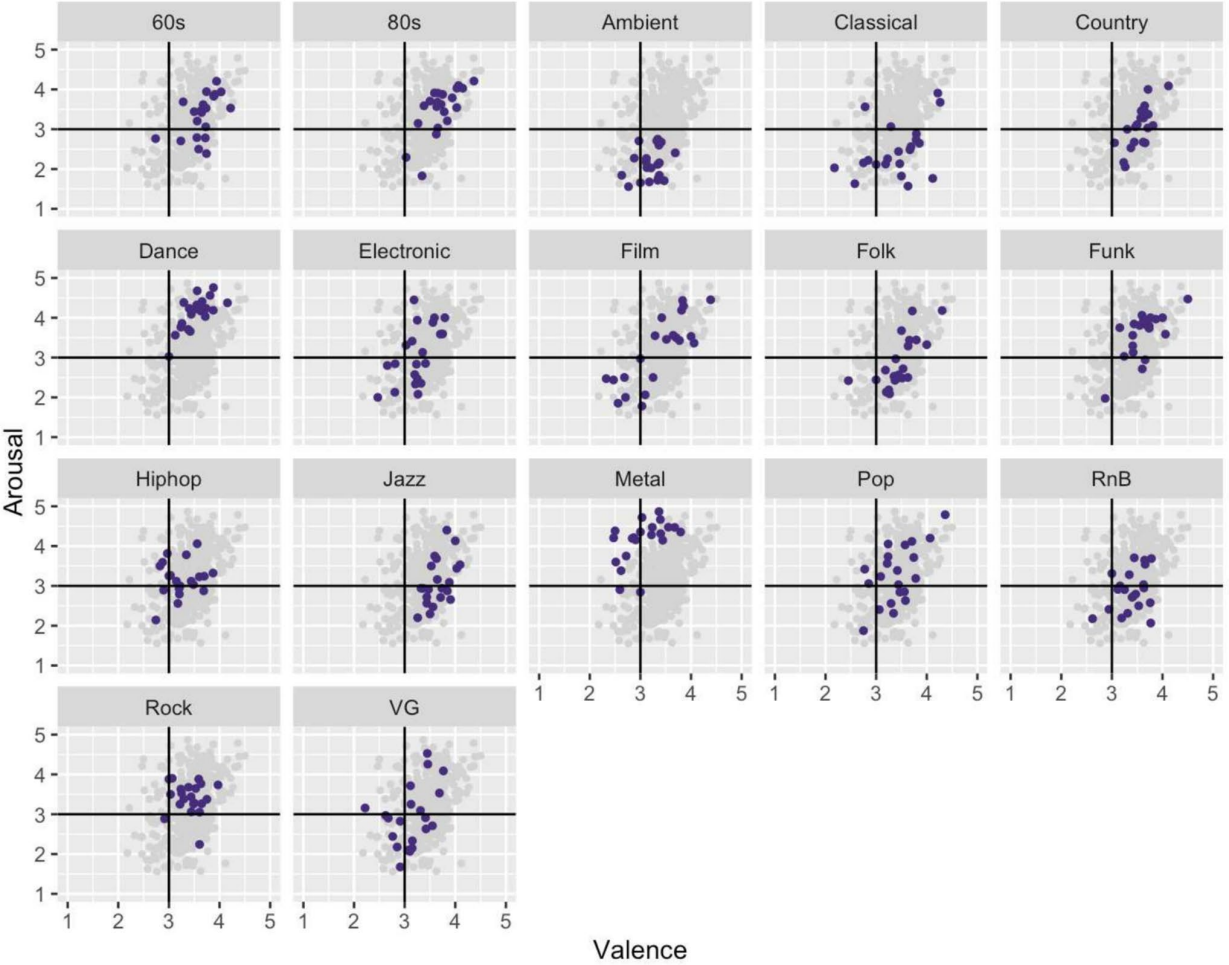
Mean emotional valence and arousal ratings of each clip by genre (VG = Video game). *Darkened points* show the clipwise mean values of each respective genre, and the *lighter clustered points* show all clipwise mean values from all genres

MUSIFEAST-17 includes normative valence and arousal ratings, enabling selection of clips for future research across a broad range of emotion space. The stimulus set covers all four quadrants of the valence and arousal space plot, with a tendency towards positive valence (see Fig. 3). Distinctly clustered genres included Ambient clips, which occupied a particularly low arousal space, Metal clips, which were typically rated as high in arousal, and Dance clips which tended to be rated both high in arousal and positive in valence. Film clips showed the widest spread in emotion space, spanning both the lower and higher ends of the valence and arousal rating ranges.

Two-way random-effects intraclass coefficients (ICC) models with a consistency-type agreement definition were used to assess inter-rater reliability for both valence and arousal ratings separately across the 21 stimulus groups (Shrout & Fleiss, 1979). All ICC values were statistically significant (*p*s < 0.05); valence ratings showed a mean ICC of 0.833 across all the stimulus groups (range = 0.472 to 0.932), and arousal ratings had a mean ICC of 0.966 (range = 0.940 to 0.986). According to interpretation guidance from Koo and Li (2016), these values indicate good overall agreement between raters’ valence scores (> 0.75) and excellent overall agreement between raters’ arousal scores (> 0.90) within each stimulus group. Valence ratings showed variability in reliability across stimulus groups, ranging from poor (< 0.50) to excellent agreement. Arousal ratings showed a high narrow range, indicating consistent excellent agreement across all stimulus groups. See Supplementary Tables 1 and 2 for a full groupwise breakdown of ICC results.

## Effects of genre, age group, and nationality on clip ratings

We aimed to provide normative data for the MUSIFEAST-17 stimulus set for US and UK listeners across adulthood by sampling evenly across the two countries and four age groups. However, we note that not all stimuli may be perceived similarly across these demographic groups and therefore analysed how these age and nationality groups impact stimulus ratings, both independently and in interaction with genre (e.g. some genres may be less familiar or less enjoyed only by certain age groups). Given that these analyses revealed some demographic differences (summarised below), alongside the normative MUSIFEAST-17 data aggregated across the full sample, we also provide eight separate summaries for each of the age groups and nationalities on our OSF repository, enabling researchers to efficiently select stimuli normed to their specific country or age range(s) of interest.

All clip ratings (clip familiarity, style familiarity, contrast, enjoyment, valence, arousal) were found to have significant positive correlations ranging from 0.097 (style familiarity with contrast) to 0.631 (enjoyment with valence) (*p*s < 0.001). This indicates these ratings were suitable as dependent variables for MANOVA testing (Cole et al., 1994). The MANOVA model, including these six clip rating variables, revealed significant effects of genre, nationality, age group, and significant interactions between genre and age group, and nationality and age group (all *p*s < 0.001). The interaction between genre and nationality was not significant (*p* = 0.308)*,* nor the three-way interaction of genre, nationality, and age group (*p* > 0.99).

For the significant effects revealed in the overall MANOVA, individual ANOVA models were fitted for each clip rating variable (see Table 1). Genre had a significant effect on all clip ratings. Post hoc analyses showed that every genre had at least one significant effect, with Eighties and Ambient music having significant effects on all six clip ratings (comparatively more and positive and negative, respectively). See Supplementary Table 3 for full post hoc analyses of genre effects.

**Table 1:** F-statistics (partial eta squared) for ANOVA results. Statistically significant effects are indicated by: p < 0.05 (*), p < 0.01 (**), p < 0.001 (***)

	Clip familiarity	Style familiarity	Contrast	Enjoyment	Valence	Arousal
*Nationality*	11.267*** (0.009)	6.158* (0.009)	0.010 (< 0.001)	20.868*** (0.031)	37.187*** (0.053)	18.833*** (0.028)
*Age group*	3.428* (0.016)	3.643* (0.016)	0.375 (0.002)	6.123*** (0.027)	4.718** (0.021)	0.439 (0.002)
*Genre*	31.907*** (0.045)	31.378*** (0.045)	19.735*** (0.029)	20.853*** (0.031)	29.591*** (0.043)	182.912*** (0.217)
Nationality*Age group	0.188 (0.008)	1.737 (0.008)	0.390 (0.002)	0.452 (0.002)	0.692 (0.003)	0.868 (0.004)
Genre*Age group	2.990*** (0.015)	3.417*** (0.015)	0.873 (0.004)	2.961*** (0.013)	2.981*** (0.013)	1.020 (0.005)

Both age group and the interaction between genre and age group had significant effects on clip familiarity, style familiarity, enjoyment, and valence ratings (not contrast or arousal). Post hoc analyses of age group revealed that the 61–75 age group gave significantly lower ratings than the 18–30 and 31–45 age groups on enjoyment, and the 61–75 age group gave significantly lower ratings than the 31–45 age group on valence (*p*s < 0.004)*.* Some genres elicited similar ratings across age groups (e.g. style familiarity and valence of Ambient music, clip familiarity and enjoyment of Classical music) but others elicited significantly different ratings across age groups (e.g. Dance and Electronic for all four significant clip ratings). Supplementary Table 4 contains the post hoc analyses results of age groups and Supplementary Figs. 1, 3, 5, and 7 display ANOVA results for the effect of age group on clip ratings across genres.

Nationality had a significant effect on all clip ratings except contrast; post-hoc analyses revealed that US participants gave significantly higher ratings than UK participants for all five significant clip ratings (*p*s < 0.013) (see Supplementary Table 5 and Supplementary Figs. 2, 4, 6, 8, 9). The interaction between nationality and age group was found to be non-significant for all clip ratings.

## Cluster analysis

K-means clustering was used to provide an exploratory summary of similar rating patterns on clip familiarity, style familiarity, contrast, enjoyment, valence, and arousal across clips within MUSIFEAST-17. The elbow method suggested three clusters were optimal; three clusters produced a lower WSS than two clusters, but adding a fourth did not result in a substantial WSS reduction. The silhouette method also suggested three clusters; three clusters produced the largest silhouette value, indicating the best balance of high within-cluster similarity to low between-cluster similarity. We therefore proceeded with three clusters for our analysis.

**Fig. 4:**
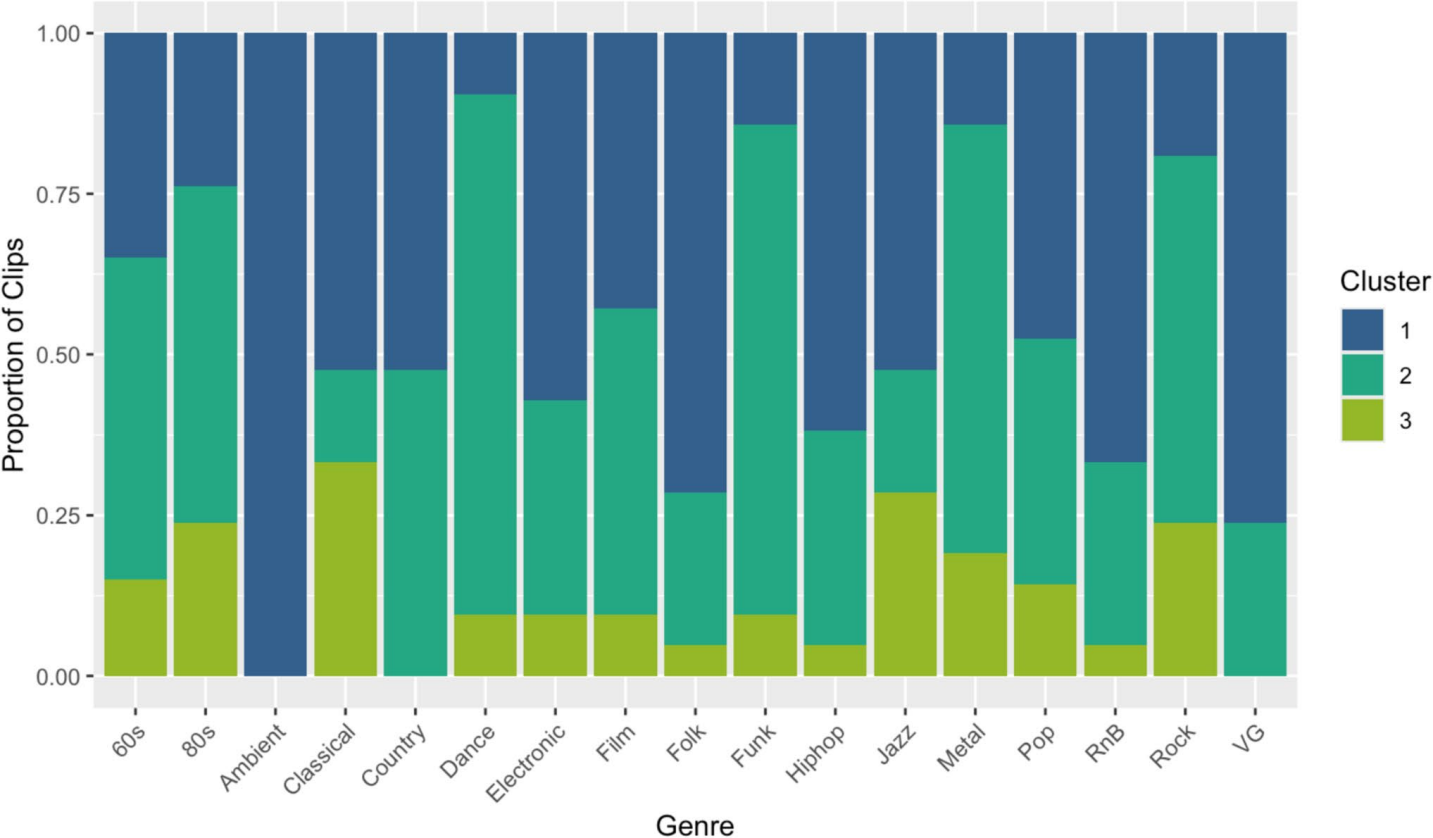
Proportion of genre clips within each cluster (VG = Video game)

As summarised in Table 2, clips in Cluster 1 were characterised by relatively low ratings for all six variables, Cluster 3 clips by relatively high ratings of five variables except arousal for which Cluster 2 clips had high ratings (and moderate ratings for the other five variables). All Ambient clips fell within Cluster 1, indicating a high degree of homogeneity in ratings within this genre and distinctiveness from the other genres (see Fig. 4). Video game, Folk, R&B, and Electronic clips fell mostly within Cluster 1, and Dance, Funk, Metal, Eighties, and Rock clips fell mostly within Cluster 2, suggesting some homogeneity in how these genres were perceived. Cluster 3 contained the fewest clips overall with no large amount of any genre’s clips classified in this cluster, although Classical music had the highest proportion of clips in Cluster 3 compared to other genres.

**Table 2:** Mean (SD) of each variable in the three clusters

Cluster	Clip familiarity	Style familiarity	Contrast	Enjoyment	Valence	Arousal
1	1.556 (0.258)	2.962 (0.402)	2.177 (0.343)	3.156 (0.382)	3.221 (0.349)	2.563 (0.439)
2	1.632 (0.272)	3.164 (0.377)	2.632 (0.527)	3.279 (0.339)	3.507 (0.350)	3.793 (0.399)
3	3.292 (0.647)	3.955 (0.318)	2.683 (0.537)	3.845 (0.263)	3.824 (0.302)	3.563 (0.771)

## Contextual associations

**Fig. 5:**
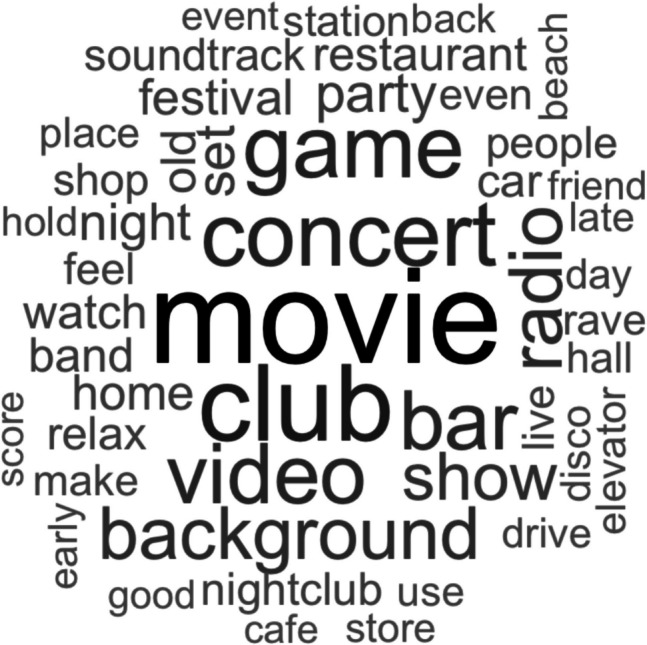
Words reported in contextual association descriptions at least 100 times over all trials for all genres. Word size corresponds to frequency of appearance (larger words = more frequent)

To provide an overview of the contexts (e.g. places, situations) linked to different genres, participants’ open-text descriptions of their contextual associations were compiled into word clouds. This novel aspect of MUSIFEAST-17 enables future studies to select stimuli that vary in their associated contexts to test how such associations impact participant evaluations and behaviours. Overall, the contexts frequently mentioned across genres were “movie”, “club”, “film”, “concert”, “game”, “bar”, and “radio” (see Fig. 5). Within each genre, there were differential patterns of contextual association, indicated by the size and number of words within each genre’s word cloud (see Appendix B for all genre word clouds). Some genres had high levels of consensus for a single contextual association, such as “movie” for Film music. Other genres showed consensus over multiple contextual associations, for example, Ambient clips were considered to be predominantly “movie”, “background”, and “(video) game” music.

## Thought types reported

**Fig. 6:**
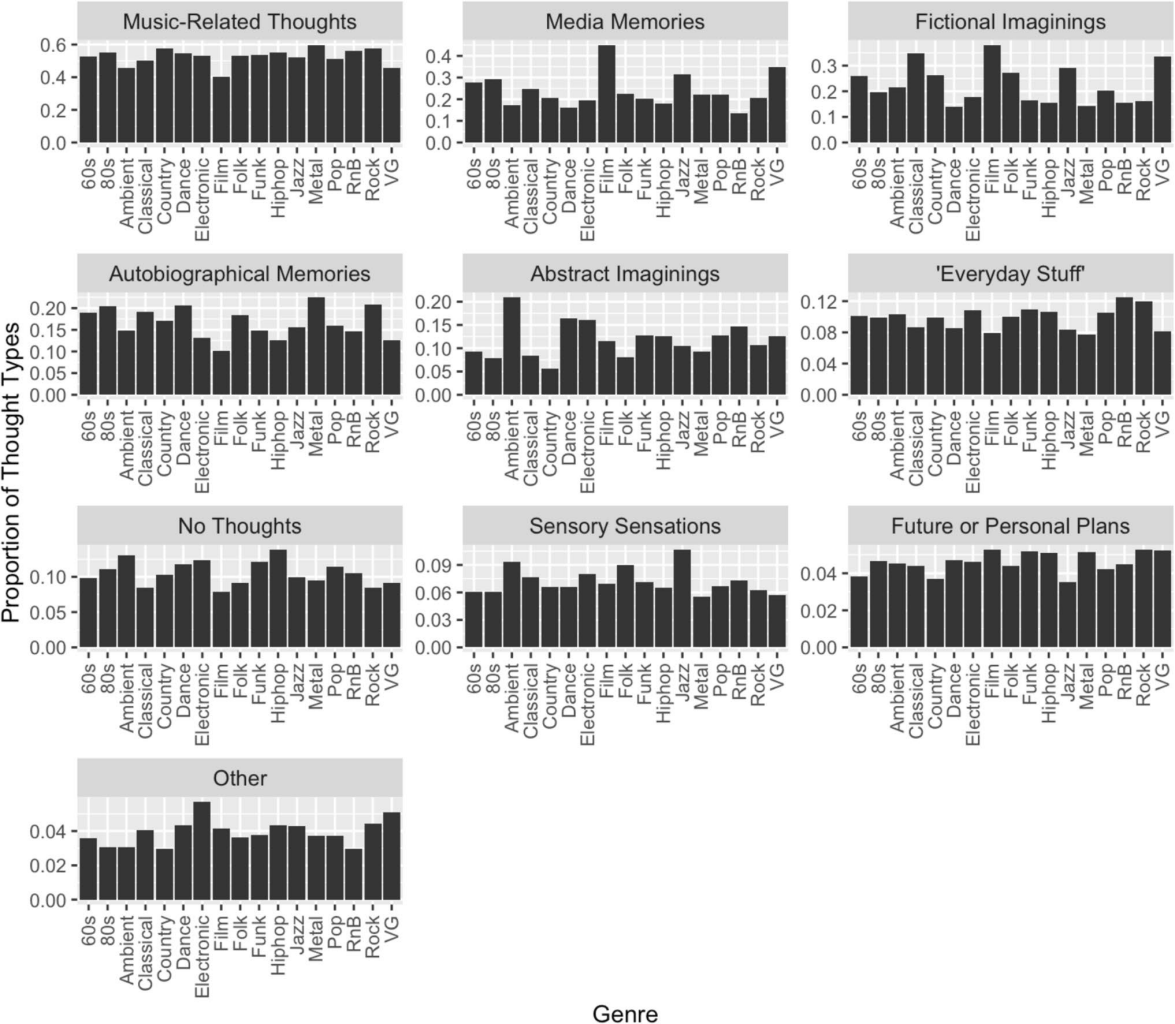
Frequency of thought types reported for each genre, summed across all trials

In 90.6% of trials, at least one thought type (i.e. not “no thoughts”) was selected from the multiple-choice list. The most frequently selected were music-related thoughts (52.6%), followed by media memories (23.6%), fictional imaginings (22.5%), autobiographical memories (16.4%), abstract imaginings (10%), no thoughts (10%), and “everyday stuff” (9%). Selections of sensory sensations (5.9%), future or personal plans (2.8%), and other thoughts (1.1%)^6^ were particularly infrequent. Figure 6 shows the frequencies of all thought types by genre.

## Discussion

This paper aimed to address a significant gap in the availability of stylistically diverse, ecologically valid music stimulus sets by developing MUSIFEAST-17. Intended to mirror the variety of musical experiences encountered in everyday life, specifically within Western (US/UK) listening contexts, our resulting stimulus set includes 356 instrumental clips from commercially released music spanning 17 genres and varied familiarity levels. The normative data for this stimulus set, collected from 701 participants from the US and UK across the range of adulthood, includes ratings on familiarity, emotional expression, genre perception, and novel contributions of contextual associations and thought types occurring during music listening.

The normative data presented in this paper indicate that we achieved our aims to compile a stimulus set of varied genres of Western music across a range of familiarity levels. Stimuli were predominantly rated as being from the USA/Canada and Europe and were perceived as being from a wide range of genres. While genre is a subjective concept and participants did not always agree with our preassigned genre labels, their ratings indicated that the stimuli represented a broad spectrum of genres. Participants used the full range of genre categories and there was frequent agreement both with the preassigned labels and among participants themselves. Participants frequently matched clips to their preassigned label from genres such as Classical, Jazz, and Ambient, which also had high levels of between-participant agreement. Dance music also displayed high levels of between-participant agreement, with nearly 80% of participants categorising the clips as either “Dance” or “Electronic”, reflecting the close relationship between these genres. Genres such as R&B, Hip-hop, and Video game music were least frequently identified, with R&B, Pop, and Hip-hop displaying the lowest levels of between-participant agreement; all of these were most frequently categorised as “Electronic” music. This pattern could be attributed to the instrumental nature of the clips, removing vocal cues that may be necessary for identifying some genres like Hip-hop (Gonzalez, 2024), and to overlapping production techniques, such as the electronic instrumentation and synthesised sounds common across these genres. While Video game clips had the fourth lowest level of participant agreement, they were most frequently rated as “Film” music, suggesting that participants could detect that the music was composed specifically for media. Although Eighties music was more often correctly identified, the majority of both Sixties and Eighties clips were identified above chance level, indicating listeners were sensitive to decade-specific stylistic cues. Our data on genre, decade and continent identification provide a strategic resource for researchers by offering a framework for exploring topics such as genre categorisation, understanding cultural and temporal influences on music perception, and developing models for music recommendation systems.

Data on genre recognition is important in music psychology because listeners’ subjective genre perceptions – regardless of alignment with objective or computational labels – significantly influence cognitive and emotional responses to music. Thompson et al.’s (2023) concept of “source sensitivity” highlights how listeners’ awareness of contextual and causal origins of music shapes their engagement and appreciation. Genre acts as a contextual cue, and is often linked to cultural and experiential associations; even in experimental contexts where excerpts may be brief and unfamiliar, genre associations can elicit specific thoughts, emotions, and memories, with empirical work showing that different genres elicit distinct thought patterns and emotional responses (Ben Sassi & Ben Yahia, 2021; Jakubowski et al., 2024; Rentfrow, 2012; Strauss et al., 2024; Zentner et al., 2008). Therefore, participant-identified genre labels provide insight into the psychological processes underpinning music listening, underscoring the need to consider subjective genre recognition as a meaningful variable in music psychology research.

Our normative data also show that the clips included in MUSIFEAST-17 vary widely in familiarity. Significant differences were found in participants’ familiarity ratings between the preassigned high, medium, and low familiarity levels, although this result varied across genres. Eleven of the 17 genres displayed significant differences between at least two of the preassigned familiarity levels, with Eighties, Jazz, and Rock clips displaying the greatest spread and significant differences across all three familiarity levels. In contrast, Country, R&B, and Video game clips did not show significant differences between any of the familiarity levels, suggesting that these genres were not as readily recognisable to participants. This may reflect either lower general exposure to these genres among the participant pool, which seems to be supported by the Genre Exposure ratings taken at the end of the study (Appendix A) for Country and Video game music, or less distinctiveness in the selected stimuli for these genres. These familiarity variations are expected when studying a broad sample of listeners across adulthood, even when constrained to Western listeners, as some genres occupy a wider range of familiarity and others may be more consistently unfamiliar. This could be due to factors such as generational differences in exposure (see Supplementary Fig. 1), the mainstream popularity of certain genres over time, or their prominence in popular media. The inclusion of genres and clips spanning different familiarity levels within MUSIFEAST-17 enables a range of research applications, such as examining emotional responses to familiar versus novel stimuli or studying how music familiarity impacts musical preferences and memory.

The musical excerpts also convey a broad range of emotions across the valence and arousal space. Some genres occupied distinct emotional areas, such as Ambient and Metal music. Film music displayed the greatest spread of emotional expression ratings, spanning both low and high ends of valence and arousal. MUSIFEAST-17 thereby enables researchers to capture natural variations in musical expression across genres and their influence on perceptual responses. Additionally, our inclusion of perceptual ratings of musical contrast (e.g. high contrast ratings for Funk and Dance versus low ratings for Ambient clips) alongside dimensions such as valence and arousal enable future exploration into which musical features underpin listener’s perceptual ratings of both contrast and emotion.

The three distinct clip clusters based on clip rating similarity across familiarity, contrast, enjoyment, valence, and arousal, helps to identify perceptual similarity within and across genres; certain genres’ clips (e.g. Classical, Ambient) were perceived more homogenously, while others (e.g. Metal, Dance), showed more variation in listener perception. By leveraging these features, MUSIFEAST-17 offers a flexible resource for researchers and those looking for tailored stimuli selection across a wide variety of musical styles. For instance, researchers could select excerpts from several different genres that fall into the same cluster if they wanted to compare the impact of music of similar familiarity, enjoyment, and emotional expression across different genres.

Our objective in this paper was to provide an overview of the normative data that highlight the unique contributions and breadth of validated dimensions included in MUSIFEAST-17. A key strength of this dataset is that researchers can make use of its dimensions both independently (e.g. focusing solely on emotional expression and selecting stimuli from specific valence and arousal quadrants) or in combination with other dimensions, enabling targeted and nuanced stimuli selection. For example, one could leverage both genre perception and contextual associations data. Electronic and Video game music share the contextual association “game”, and Dance and Electronic music share the contextual association “club”; both pairs were often labelled as the same genre. However, Funk music, predominantly associated as “club” music, was not perceived as particularly similar to either Dance or Electronic music. Researchers could use genre perception and contextual association data to select stimuli with differing or matching genre labels combined with shared imagined contexts to investigate whether contextual associations or genre labels primarily drive listener responses. For example, listener responses such as emotional reactions, memory recall, or genre-based expectations might be influenced by whether the context or the given genre label aligns with their perception of the music. Other such usage of the validated dimensions of MUSIFEAST-17 can enable future studies investigating how interconnected musical features shape perception.

The limitations of MUSIFEAST-17 include its focus on Western genres, as the stimuli were informed by UK and US listening statistics and participant recruitment was limited to these geopolitical regions. Individual demographic differences (e.g. age groups) were also expected and acknowledged, having sampled across the range of adulthood up to age 75. The oldest age group found the stimulus set more unfamiliar than the other age groups, likely because the genre and stimuli selection were based on listening statistics from 2024. However, some genres did not show this effect, suggesting that users could selectively choose genres that are similarly intergenerationally familiar if the study design requires minimizing familiarity-based differences across age groups. Additionally, US participants consistently rated the clips higher across all measurements, perhaps due to different cultural attitudes between nationalities or, similarly to the age group differences, the way in which the stimuli were collected (e.g. Film stimuli chosen from IMDb’s movies ranked by US box office). These differences in perception of the stimulus set are explored in the Results section and separate normed data specific to these individual demographic groups (eight additional datasets, split by nationality and age group) are provided in the MUSIFEAST-17 repository.

While MUSIFEAST-17 provides a notable range of stimuli for Western listeners, future expansions could broaden its applicability. Future iterations of this stimulus set could incorporate non-Western and other currently underrepresented genres in this domain and ratings from non-Western listeners. These could enable cross-cultural research and address how cultural context influences music perception and engagement. Including stimuli that capture hybrid genres, such as contemporary fusion styles, would further enhance the ecological validity of the set. Additionally, incorporating longer versions of some clips (1 min or more) could allow for the exploration of more dynamic responses or ones that unfold over a longer timescale. The integration of additional metadata (e.g. tempo, key, instrumentation) could facilitate inquiries into the acoustic and structural elements of music that drive listener responses. The open-access format of the MUSIFEAST-17 repository encourages collaboration and feedback from the research community, enabling the set to evolve and expand to meet emerging needs, address limitations, and support innovative studies into the cognitive, emotional, and social dimensions of musical experience.

## Supplementary Information

Below is the link to the electronic supplementary material.

Supplementary file1 (DOCX 3948 KB)

## Data Availability

The datasets generated and analysed during the current study, including all music stimuli and Supplementary Material, are available at https://osf.io/5ebz2/.

## Appendix A: Survey questions

***Clip response questions:***

[*presented after each clip*].

## Appendix B: Word cloud plots

Word cloud plots of terms reported in contextual association descriptions at least 15 times over all trials for each individual genre. Word size corresponds to the frequency of appearance (larger words = more frequent).

Common stopwords removed using tm_map(corpus, removeWords, stopwords("en")) https://rdrr.io/rforge/tm/man/stopwords.html

Custom stopwords: "music","thought","think","imagine","scene","song","made","around","listen","sound","remind","like","play","sort","that","much","decide","something","just","also","unsure","yes","yea","none","nothing","non","please","actually","didn’t","initially","maybe","sure","associate","time","can","type","setting","hear","see","expect","used","never","perhaps","probably","kind","got","one","pattern","etc","several","anything","honestly","banana","cant","tell","lol","guess","sixty","60s","1960s","1960","eighty","80s","1980s","1980","ambient","classical","classic","country","dance","electronic","film","folk","funk","hiphop","jazz","metal","pop","rock","rnb","videogame"

## Footnotes

1 https://www.kaggle.com/datasets/andradaolteanu/gtzan-dataset-music-genre-classification/data

2 90 of the 356 clips contain these vocables. Specifics are listed in the “MUSIFEAST-17 Normative Data Tables” and “MUSIFEAST17_metadata” file accessed via the OSF project link.

3 “Top artists” proved to be a more stable and diverse representation of each genre, whereas “Top clips” were dominated by a few artists and changed frequently, making them unreliable for our purposes.

4 For genre identification, responses were scored as correct if participants selected the respective genre in either the primary or secondary genre question in the “Clip Response” survey (see Appendix B). Sixties and Eighties music were not considered in this analysis, but correct identification of the decade of release for these subgenres was examined in subsequent analyses.

5 Creation decade rating data for all other genres, alongside release dates and decade identification accuracy rates, is included in the MUSIFEAST-17 dataset online repository on OSF.

6 Examples of responses in the “Other” thought types text box: “Made me want to move with the music.”, “I was waiting for the music to pick up”, “I found it annoying.”, “It was more feeling than thought: fun, youth, carefree”, “Musicianship—I was deciding if the saxophone player was skillful”.

## Code availability

All analyses were conducted in *RStudio* (version 2024.04.1 + 748). The code used in this study is available at https://osf.io/5ebz2/.

## Declarations

### Open practices statement

All music stimuli, validation data, datasets generated and analysed during the current study, Supplementary Material, and analysis code are openly available online in the MUSIFEAST-17 repository hosted by OSF at https://osf.io/5ebz2/. The reported study was not preregistered.

## References

[CR1] Aljanaki, A., Kalonaris, S., Micchi, G., & Nichols, E. (2021). MCMA: A Symbolic Multitrack Contrapuntal Music Archive. *Empirical Musicology Review, 16*(1), 99–105. 10.18061/emr.v16i1.7637

[CR2] Aljanaki, A., Yang, Y. H., & Soleymani, M. (2017). Developing a benchmark for emotional analysis of music. *PLoS ONE,**12*(3), Article e0173392. 28282400 10.1371/journal.pone.0173392 PMC5345802

[CR3] Baker, D. J. (2021). *MeloSol Corpus. Empirical Musicology Review,**16*(1), 106–113.

[CR4] Belfi, A. M., & Kacirek, K. (2021). The famous melodies stimulus set. *Behavior Research Methods,**53*, 34–48. 32556964 10.3758/s13428-020-01411-6

[CR5] Ben Sassi, I., & Ben Yahia, S. (2021). How does context influence music preferences: A user-based study of the effects of contextual information on users’ preferred music. *Multimedia Systems,**27*(2), 143–160.

[CR6] Cohen, J. (1988). *Statistical Power Analysis for the**Behavioral**Sciences* (2nd ed.). Routledge. 10.4324/9780203771587

[CR7] Cole, D. A., Maxwell, S. E., Arvey, R., & Salas, E. (1994). How the power of MANOVA can both increase and decrease as a function of the intercorrelations among the dependent variables. *Psychological Bulletin,**115*(3), 465.

[CR8] Darcy, I., & Fontaine, N. M. G. (2020). The Hoosier Vocal Emotions Corpus: A validated set of North American English pseudo-words for evaluating emotion processing. *Behavior Research Methods,**52*, 901–917. 10.3758/s13428-019-01288-0 31485866 10.3758/s13428-019-01288-0

[CR9] De Deyne, S., Navarro, D. J., Perfors, A., Brysbaert, M., & Storms, G. (2019). The “Small World of Words” English word association norms for over 12,000 cue words. *Behavior Research Methods,**51*, 987–1006. 10.3758/s13428-018-1115-7 30298265 10.3758/s13428-018-1115-7

[CR10] Eerola, T., & Vuoskoski, J. K. (2011). A comparison of the discrete and dimensional models of emotion in music. *Psychology of Music,**39*(1), 18–49. 10.1177/0305735610362821

[CR11] Eschrich, S., Münte, T.F. & Altenmüller, E.O. (2008). Unforgettable film music: The role of emotion in episodic long-term memory for music. *BMC Neuroscience, 9*(48). 10.1186/1471-2202-9-48 10.1186/1471-2202-9-48 PMC2430709 18505596

[CR12] Gonzalez, L. (2024). Corpus-Based Studies on Hip-Hop Lyrics. (https://ir.library.oregonstate.edu/concern/graduate_thesis_or_dissertations/0r967c68d). Accessed 24 Dec 2024

[CR13] Hosken, F., Bechtold, T., Hoesl, F., Kilchenmann, L., & Senn, O. (2021). Drum Groove Corpora. *Empirical Musicology Review, 16*(1), 114–123. 10.18061/emr.v16i1.7642

[CR15] Jakubowski, K., Margulis, E. H., & Taruffi, L. (2024). Music-evoked thoughts: Genre and emotional expression of music impact concurrent imaginings. *Music Perception,**42*(1), 3–18. 10.1525/mp.2024.42.1.3

[CR16] Kirchberger, M., & Russo, F. A. (2016). Dynamic range across music genres and the perception of dynamic compression in hearing-impaired listeners. *Trends in Hearing,**20*, 2331216516630549. 26868955 10.1177/2331216516630549 PMC4753356

[CR17] Koelsch, S., Gunter, T. C., Wittfoth, M., & Sammler, D. (2005). Interaction between syntax processing in language and in music: An ERP study. *Journal of Cognitive Neuroscience,**17*(10), 1565–1577. 10.1162/089892905774597290 16269097 10.1162/089892905774597290

[CR18] Koelsch, S., Skouras, S., Fritz, T., Herrera, P., Bonhage, C., Küssner, M. B., & Jacobs, A. M. (2013). The roles of superficial amygdala and auditory cortex in music-evoked fear and joy. *NeuroImage,**1*(81), 49–60. 10.1016/j.neuroimage.2013.05.008 10.1016/j.neuroimage.2013.05.008 23684870

[CR19] Koo, T. K., & Li, M. Y. (2016). A guideline of selecting and reporting intraclass correlation coefficients for reliability research. *Journal of Chiropractic Medicine,**15*(2), 155–163. 10.1016/j.jcm.2016.02.012 27330520 10.1016/j.jcm.2016.02.012 PMC4913118

[CR20] Lassalle, A., Pigat, D., O’Reilly, H., Fridenson-Hayo, S., Tal, S., Elfström, S., Råde, A., Golan, O., Bölte, S., Baron-Cohen, S., & Lundqvist, D. (2019). The EU-Emotion Voice Database. *Behavior Research Methods,**51*, 493–506. 29713953 10.3758/s13428-018-1048-1 PMC6478635

[CR21] Livingstone, S. R., & Russo, F. A. (2018). The Ryerson Audio-Visual Database of Emotional Speech and Song (RAVDESS): A dynamic, multimodal set of facial and vocal expressions in North American English. *PLoS ONE,**13*(5), Article e0196391. 10.1371/journal.pone.0196391 29768426 10.1371/journal.pone.0196391 PMC5955500

[CR22] Llorens, A. (2021). The Recorded Brahms Corpus (RBC): A Dataset of Performative Parameters in Recordings of Brahms’s Cello Sonatas. *Empirical Musicology Review, 16*(1), 124–133. 10.18061/emr.v16i1.7612

[CR23] Loui, P., Kubit, B. M., Ou, Y., & Margulis, E. H. (2023). Imaginings from an unfamiliar world: Narrative engagement with a new musical system. *Psychology of Aesthetics, Creativity, and the Arts*.

[CR24] Margulis, E. H., & Jakubowski, K. (2024). Music, memory, and imagination. *Current Directions in Psychological Science,**33*(2), 108–113.

[CR26] McKay, C., McEnnis, D., & Fujinaga, I. (2006). A Large Publicly Accessible Prototype Audio Database for Music Research. In *Proceedings of the 20th Int. Soc. for Music Information Retrieval Conference (ISMIR)*, 160–163.

[CR27] Murthy, Y. S., & Koolagudi, S. G. (2018). Content-based music information retrieval (CB-MIR) and its applications toward the music industry: A review. *ACM Computing Surveys (CSUR),**51*(3), 1–46.

[CR28] Ollen, J. E. (2006). A criterion-related validity test of selected indicators of musical sophistication using expert ratings [electronic resource]. http://www.ohiolink.edu/etd/view.cgi?osu1161705351. Accessed Jan 2024.

[CR29] Peirce, J. W., Gray, J. R., Simpson, S., MacAskill, M. R., Höchenberger, R., Sogo, H., Kastman, E., Lindeløv, J. (2019). PsychoPy2: experiments in behavior made easy. *Behavior Research Methods* 51, 195–203 (2019). 10.3758/s13428-018-01193-y. 10.3758/s13428-018-01193-y PMC6420413 30734206

[CR30] Peretz, I., Babaï, M., Lussier, I., Hebert, S., & Gagnon, L. (1995). Corpus d’extraits musicaux: Indices relatifs à la familiarité, à l’âge d’acquisition et aux évocations verbales. / A repertory of music extracts: Indicators of familiarity, age of acquisition, and verbal associations. *Revue Canadienne De Psychologie Expérimentale / Canadian Journal of Experimental Psychology,**49*, 211–239. 10.1037/1196-1961.49.2.211 9221056

[CR31] Posit team (2025). *RStudio: Integrated Development Environment for R*. Posit Software, PBC. http://www.posit.co/. Accessed June 2025.

[CR32] Prolific (2023). London, UK. https://www.prolific.com. Accessed Mar 2024.

[CR33] Rainsford, M., Palmer, M. A., & Paine, G. (2018). The MUSOS (MUsic SOftware System) Toolkit: A computer-based, open-source application for testing memory for melodies. *Behavior Research Methods,**50*, 684–702. 10.3758/s13428-017-0894-6 28432568 10.3758/s13428-017-0894-6

[CR34] Rentfrow, P. J. (2012). The role of music in everyday life: Current directions in the social psychology of music. *Social and Personality Psychology Compass,**6*(5), 402–416.

[CR35] Scott, G. G., Keitel, A., Becirspahic, M., Yao, B., & Sereno, S. C. (2019). The Glasgow Norms: Ratings of 5,500 words on nine scales. *Behavior Research Methods,**51*, 1258–1270. 30206797 10.3758/s13428-018-1099-3 PMC6538586

[CR36] Shrout, P. E., & Fleiss, J. L. (1979). Intraclass correlations: Uses in assessing rater reliability. *Psychological Bulletin,**86*(2), 420. 18839484 10.1037//0033-2909.86.2.420

[CR37] Srinivasamurthy, A., Gulati, S., Caro Repetto, R., & Serra, X. (2021). Saraga: Open Datasets for Research on Indian Art Music. *Empirical Musicology Review, 16*(1), 85–98. 10.18061/emr.v16i1.7641

[CR38] Standard Repertoire (2021). The Top 25 Jazz Standards. https://standardrepertoire.com/pages/the-top-25-jazz-standards.html. Accessed 4 Feb 2024.

[CR39] Statista (2024). Preferred radio content by genre in the UK 2023. https://www.statista.com/forecasts/997920/preferred-radio-content-by-genre-in-the-uk. Accessed Oct 2023.

[CR40] Statista (2024b). Streamed music consumption in the U.S. by genre 2021. https://www.statista.com/statistics/475667/streamed-music-consumption-genre-usa/. Accessed Oct 2023.

[CR41] Strauss, H., Vigl, J., Jacobsen, P. O., Bayer, M., Talamini, F., Vigl, W., Zangerle, E., & Zentner, M. (2024). The Emotion-to-Music Mapping Atlas (EMMA): A systematically organized online database of emotionally evocative music excerpts. *Behavior Research Methods,**56*(4), 3560–3577. 10.3758/s13428-024-02336-0 10.3758/s13428-024-02336-0 PMC11133078 38286947

[CR42] Tzanetakis, G., & Cook, P. (2002). Musical genre classification of audio signals. *IEEE Transactions on Speech and Audio Processing,**10*(5), 293–302.

[CR43] Thompson, W. F., Bullot, N. J., & Margulis, E. H. (2023). The psychological basis of music appreciation: Structure, self, source. *Psychological Review,**130*(1), 260. 35420849 10.1037/rev0000364

[CR44] Vieillard, S., Peretz, I., Gosselin, N., Khalfa, S., Gagnon, L., & Bouchard, B. (2008). Happy, sad, scary and peaceful musical excerpts for research on emotions. *Cognition and Emotion,**22*(4), 720–752. 10.1080/02699930701503567

[CR45] Warrenburg, L. A. (2021). The PUMS Database: A corpus of previously-used musical stimuli in 306 studies of music and emotion. *Empirical Musicology Review, 16*(1), 145–150. 10.18061/emr.v16i1.7208

[CR46] Warrenburg, L. A. (2020). Choosing the right tune: A review of music stimuli used in emotion research. *Music Perception,**37*(3), 240–258.

[CR47] White, C. W., & Quinn, I. (2016). The Yale-Classical Archives Corpus. *Empirical Musicology Review*, *11*(1), 50–58. 10.18061/emr.v11i1.4958

[CR48] Williams, J., & Sachs, M. (2023). Combating Reductionism in Music Neuroscience with Ecologically Valid Paradigms: What Can (and Cannot) Be Gained? In E. H. Margulis, P. Loui, & D. Loughridge (Eds.), *The Science-Music Borderlands: Reckoning with the Past and Imagining the Future* (pp. 239–262). MIT Press.

[CR49] Yesiler, F., Tralie, C., Correya, A., Silva, D. F., Tovstogan, P., Gómez. E., & Serra, X. (2019). Da-TACOS: A Dataset for Cover Song Identification and Understanding. In *Proceedings of the 20th Int. Soc. for Music Information Retrieval Conference**(ISMIR)*, 327–334.

[CR50] Zentner, M., Grandjean, D., & Scherer, K. R. (2008). Emotions evoked by the sound of music: Characterization, classification, and measurement. *Emotion,**8*(4), 494. 18729581 10.1037/1528-3542.8.4.494

