# Supplementary material for: MUSIFEAST-17: MUsic Stimuli for imagination, familiarity, emotion, and Aesthetic STudies across 17 genres
Source: Behav Res Methods. 2025 Jun 20;57(7):204. doi: 10.3758/s13428-025-02724-0 (PMC12181142; doi:10.3758/s13428-025-02724-0)

Supplementary material:

*Supplementary Table 1. Intraclass coefficient (ICC) values (two-way random-effects models with a consistency-type agreement definition) for valence ratings across the 21 stimulus groups.*

| **Stimulus_Group** | **ICC_Value** | **Lower_CI** | **Upper_CI** | **Clips** | **Raters** | **F_Value** | **P_Value** |
| --- | --- | --- | --- | --- | --- | --- | --- |
| ***1*** | 0.900 | 0.817 | 0.957 | 17 | 34 | 10.01 | 3.27E-22 |
| ***2*** | 0.804 | 0.641 | 0.916 | 17 | 34 | 5.10 | 6.16E-10 |
| ***3*** | 0.727 | 0.501 | 0.883 | 17 | 34 | 3.66 | 2.18E-06 |
| ***4*** | 0.918 | 0.850 | 0.965 | 17 | 34 | 12.19 | 2.02E-27 |
| ***5*** | 0.737 | 0.511 | 0.891 | 16 | 34 | 3.81 | 1.99E-06 |
| ***6*** | 0.932 | 0.876 | 0.971 | 17 | 34 | 14.74 | 2.6E-33 |
| ***7*** | 0.472 | 0.035 | 0.773 | 17 | 35 | 1.89 | 1.87E-02 |
| ***8*** | 0.918 | 0.850 | 0.965 | 17 | 32 | 12.22 | 3.21E-27 |
| ***9*** | 0.891 | 0.802 | 0.953 | 17 | 38 | 9.20 | 1.66E-20 |
| ***10*** | 0.903 | 0.822 | 0.958 | 17 | 32 | 10.28 | 1.17E-22 |
| ***11*** | 0.871 | 0.764 | 0.945 | 17 | 34 | 7.75 | 1.3E-16 |
| ***12*** | 0.909 | 0.834 | 0.961 | 17 | 32 | 11.04 | 1.88E-24 |
| ***13*** | 0.847 | 0.720 | 0.934 | 17 | 32 | 6.53 | 1.88E-13 |
| ***14*** | 0.846 | 0.718 | 0.934 | 17 | 32 | 6.48 | 2.47E-13 |
| ***15*** | 0.912 | 0.839 | 0.962 | 17 | 35 | 11.33 | 1.69E-25 |
| ***16*** | 0.816 | 0.662 | 0.921 | 17 | 31 | 5.42 | 1.14E-10 |
| ***17*** | 0.889 | 0.797 | 0.953 | 17 | 31 | 9.04 | 1.45E-19 |
| ***18*** | 0.896 | 0.811 | 0.955 | 17 | 38 | 9.64 | 1.36E-21 |
| ***19*** | 0.754 | 0.550 | 0.895 | 17 | 30 | 4.07 | 2.59E-07 |
| ***20*** | 0.878 | 0.776 | 0.948 | 17 | 33 | 8.17 | 1.36E-17 |
| ***21*** | 0.666 | 0.390 | 0.857 | 17 | 32 | 3.00 | 8.37E-05 |

*Supplementary Table 2. Intraclass coefficient (ICC) values (two-way random-effects models with a consistency-type agreement definition) for arousal ratings across the 21 stimulus groups.*

| **Stimulus_Group** | **ICC_Value** | **Lower_CI** | **Upper_CI** | **Clips** | **Raters** | **F_Value** | **P_Value** |
| --- | --- | --- | --- | --- | --- | --- | --- |
| ***1*** | 0.947 | 0.903 | 0.977 | 17 | 34 | 18.92 | 1.89E-42 |
| ***2*** | 0.975 | 0.954 | 0.989 | 17 | 34 | 39.61 | 1.1E-79 |
| ***3*** | 0.972 | 0.949 | 0.988 | 17 | 34 | 36.15 | 3.27E-74 |
| ***4*** | 0.972 | 0.948 | 0.988 | 17 | 34 | 35.30 | 8E-73 |
| ***5*** | 0.962 | 0.930 | 0.984 | 16 | 34 | 26.57 | 3.82E-54 |
| ***6*** | 0.980 | 0.964 | 0.992 | 17 | 34 | 50.80 | 8.64E-96 |
| ***7*** | 0.979 | 0.961 | 0.991 | 17 | 35 | 47.46 | 3.48E-92 |
| ***8*** | 0.956 | 0.920 | 0.981 | 17 | 32 | 22.88 | 9.78E-50 |
| ***9*** | 0.970 | 0.946 | 0.987 | 17 | 38 | 33.68 | 1.56E-72 |
| ***10*** | 0.968 | 0.942 | 0.986 | 17 | 32 | 31.73 | 1.34E-65 |
| ***11*** | 0.953 | 0.914 | 0.980 | 17 | 34 | 21.31 | 2.27E-47 |
| ***12*** | 0.944 | 0.897 | 0.976 | 17 | 32 | 17.82 | 1.38E-39 |
| ***13*** | 0.978 | 0.960 | 0.991 | 17 | 32 | 45.63 | 1.18E-86 |
| ***14*** | 0.975 | 0.955 | 0.989 | 17 | 32 | 40.77 | 9.6E-80 |
| ***15*** | 0.969 | 0.944 | 0.987 | 17 | 35 | 32.58 | 6.73E-69 |
| ***16*** | 0.959 | 0.924 | 0.982 | 17 | 31 | 24.23 | 7.5E-52 |
| ***17*** | 0.951 | 0.911 | 0.979 | 17 | 31 | 20.51 | 9.79E-45 |
| ***18*** | 0.977 | 0.958 | 0.990 | 17 | 38 | 43.63 | 4.33E-89 |
| ***19*** | 0.986 | 0.974 | 0.994 | 17 | 30 | 69.74 | 2.59E-112 |
| ***20*** | 0.982 | 0.967 | 0.992 | 17 | 33 | 55.03 | 3.84E-100 |
| ***21*** | 0.940 | 0.891 | 0.974 | 17 | 32 | 16.73 | 2.85E-37 |

*Supplementary Table 3. Contrast estimate (SE) values comparing EMM values from post-hoc analyses for the significant effect of genre on all six rating variables. Significant results (Bonferroni corrected for 17 tests, p<0.003) indicated by asterisks (*).*

| ***Genre*** | **Clip familiarity** | **Style familiarity** | **Contrast** | **Enjoyment** | **Valence** | **Arousal** |
| --- | --- | --- | --- | --- | --- | --- |
| ***Sixties pop*** | 0.059 (.041) | -0.023 (.037) | 0.128 (.037) | 0.182 (.039)* | 0.233 (.033)* | 0.137 (.035)* |
| ***Eighties pop*** | 0.340 (.055)* | 0.251 (.043)* | 0.162 (.035)* | 0.227 (.041)* | 0.305 (.033)* | 0.302 (.037)* |
| ***Ambient*** | -0.324 (.029)* | -0.345 (.037)* | -0.458 (.031)* | -0.190 (.042)* | -0.215 (.034)* | -1.071 (.033)* |
| ***Classical*** | 0.573 (.029)* | 0.333 (.038)* | -0.170 (.035)* | 0.190 (.041)* | -0.032 (.038) | -0.742 (.038)* |
| ***Country*** | -0.186 (.053)* | 0.065 (.035) | -0.045 (.032) | 0.158 (.037)* | 0.140 (.031)* | -0.086 (.034) |
| ***Dance*** | -0.128 (.029) | 0.019 (.036) | -0.046 (.038) | -0.150 (.042) | 0.088 (.033) | 0.904 (.031)* |
| ***Electronic*** | -0.119 (.039) | -0.326 (.040)* | 0.019 (.037) | -0.351 (.041)* | -0.154 (.033)* | -0.097 (.039) |
| ***Film*** | 0.123 (.044) | 0.082 (.038) | 0.095 (.036) | 0.144 (.038)* | -0.091 (.038) | -0.090 (.044) |
| ***Folk*** | -0.043 (.042) | -0.159 (.0396)* | -0.201 (.034)* | 0.081 (.040) | 0.053 (.032) | -0.296 (.036)* |
| ***Funk*** | -0.070 (.040) | -0.105 (.037) | 0.101 (.035) | 0.063 (.040) | 0.200 (.032)* | 0.364 (.034)* |
| ***Hip-hop*** | -0.219 (.034)* | -0.219 (.039)* | 0.044 (.040) | -0.321 (.040)* | -0.170 (.031)* | -0.022 (.034) |
| ***Jazz*** | 0.296 (.050)* | 0.203 (.037)* | 0.010 (.035) | 0.268 (.040)* | 0.256 (.032)* | -0.078 (.037) |
| ***Metal*** | 0.109 (.044) | 0.355 (.040)* | 0.238 (.038)* | -0.124 (.047) | -0.354 (.040)* | 1.007 (.033)* |
| ***Pop*** | -0.055 (.041) | -0.061 (.039) | -0.020 (.036) | -0.067 (.039) | -0.019 (.032) | 0.108 (.039) |
| ***R&B*** | -0.234 (.034)* | -0.201 (.036)* | 0.114 (.040) | -0.102 (.039) | -0.020 (.031) | -0.308 (.036)* |
| ***Rock*** | 0.104 (.042) | 0.240 (.039)* | 0.075 (.033) | 0.056 (.039) | 0.010 (.030) | 0.226 (.033)* |
| ***Video game*** | 0.226 (.032)* | -0.108 (.038) | -0.046 (.032) | -0.066 (.039) | -0.229 (.035)* | -0.257 (.043)* |

*Supplementary Table 4. Contrast estimate (SE) values comparing EMM values from post-hoc analyses for the significant effect of age group on the four rating variables with significant ANOVA results. Significant results (Bonferroni corrected for 6 tests, p<.008) indicated by asterisks (*).*

| ***Age group*** | **Clip familiarity** | **Style familiarity** | **Enjoyment** | **Valence** |
| --- | --- | --- | --- | --- |
| ***18to30 – 31to45*** | 0.056 (.073) | -0.176 (.077) | -0.036 (.070) | -0.065 (.048) |
| ***18to30 – 46to60*** | 0.046 (.073) | -0.181 (.077) | 0.052 (.071) | -0.019 (.049) |
| ***18to30 – 61to75*** | 0.221 (.073) | 0.003 (.077) | 0.244 (.071)* | 0.112 (.049) |
| ***31to45 – 46to60*** | -0.010 (.073) | -0.005 (.077) | 0.088 (.071) | 0.046 (.049) |
| ***31to45 – 61to75*** | 0.165 (.073) | 0.179 (.077) | 0.280 (.071)* | 0.177 (.049)* |
| ***46to60 – 61to75*** | 0.1752 (.074) | 0.185 (.078) | 0.192 (.071) | 0.131 (.049) |

*Supplementary Table 5. Contrast estimate (SE) values comparing EMM values from post-hoc analyses for the significant effect of nationality on the five rating variables with significant ANOVA results. Significant results (p<.05) indicated by asterisks (*).*

| ***Nationality*** | **Clip familiarity** | **Style familiarity** | **Enjoyment** | **Valence** | **Arousal** |
| --- | --- | --- | --- | --- | --- |
| ***UK - US*** | -0.174 (.052)* | -0.135 (.055)* | -0.230 (.050)* | -0.210 (.034)* | -0.145 (.034)* |

*Supplementary Figure 1. ANOVA plot for the effect of age group on clip familiarity ratings across genres*
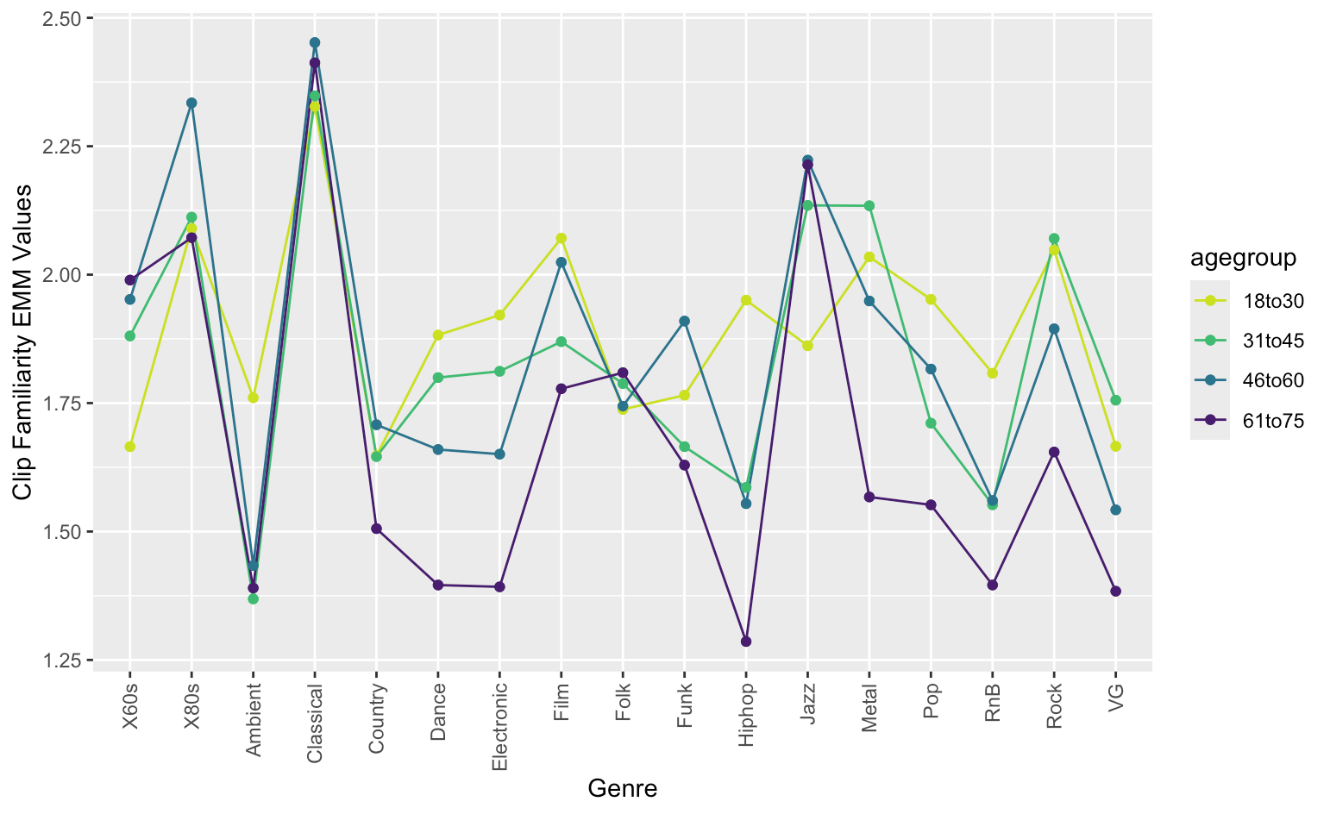


*Supplementary Figure 2. ANOVA plot for the effect of nationality on clip familiarity ratings across genres*
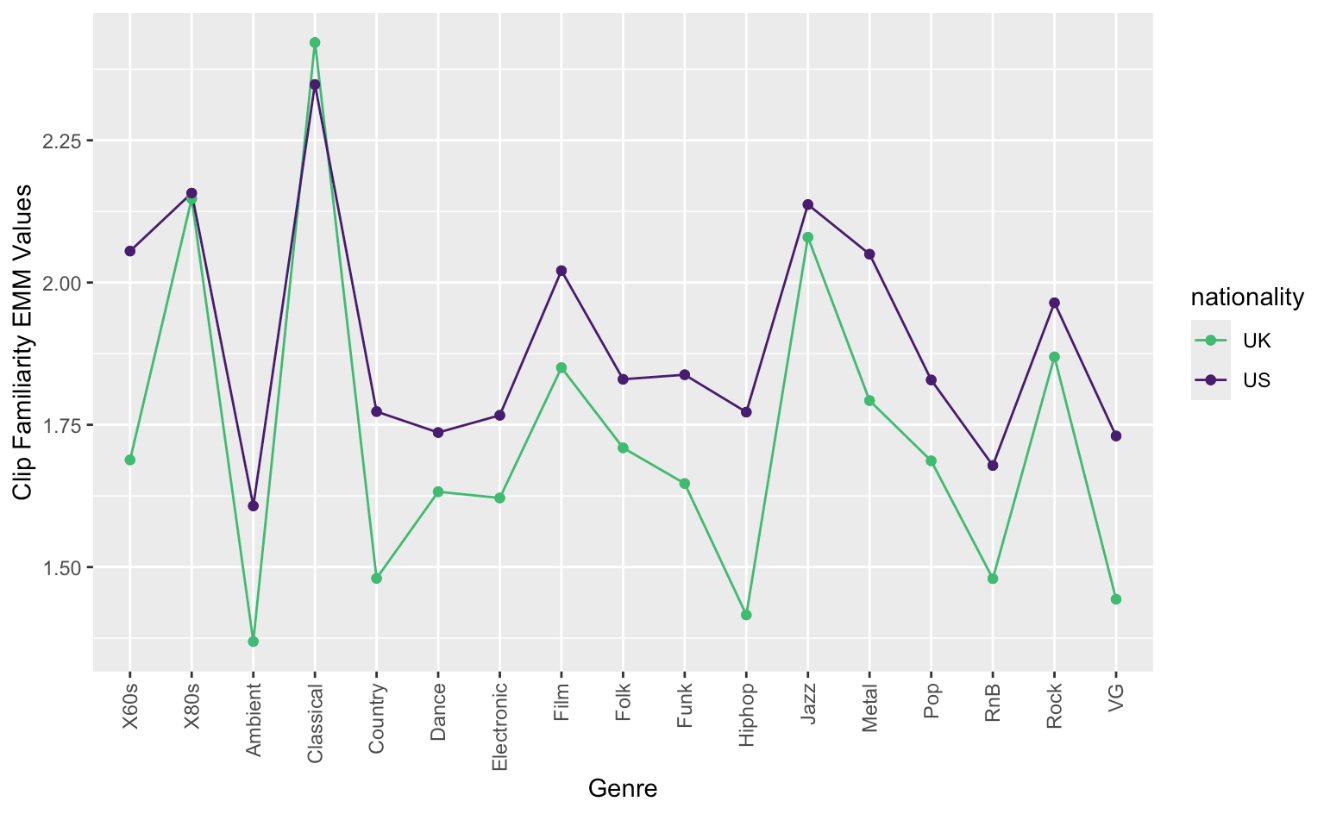


*Supplementary Figure 3. ANOVA plot for the effect of age group on style familiarity ratings across genres*
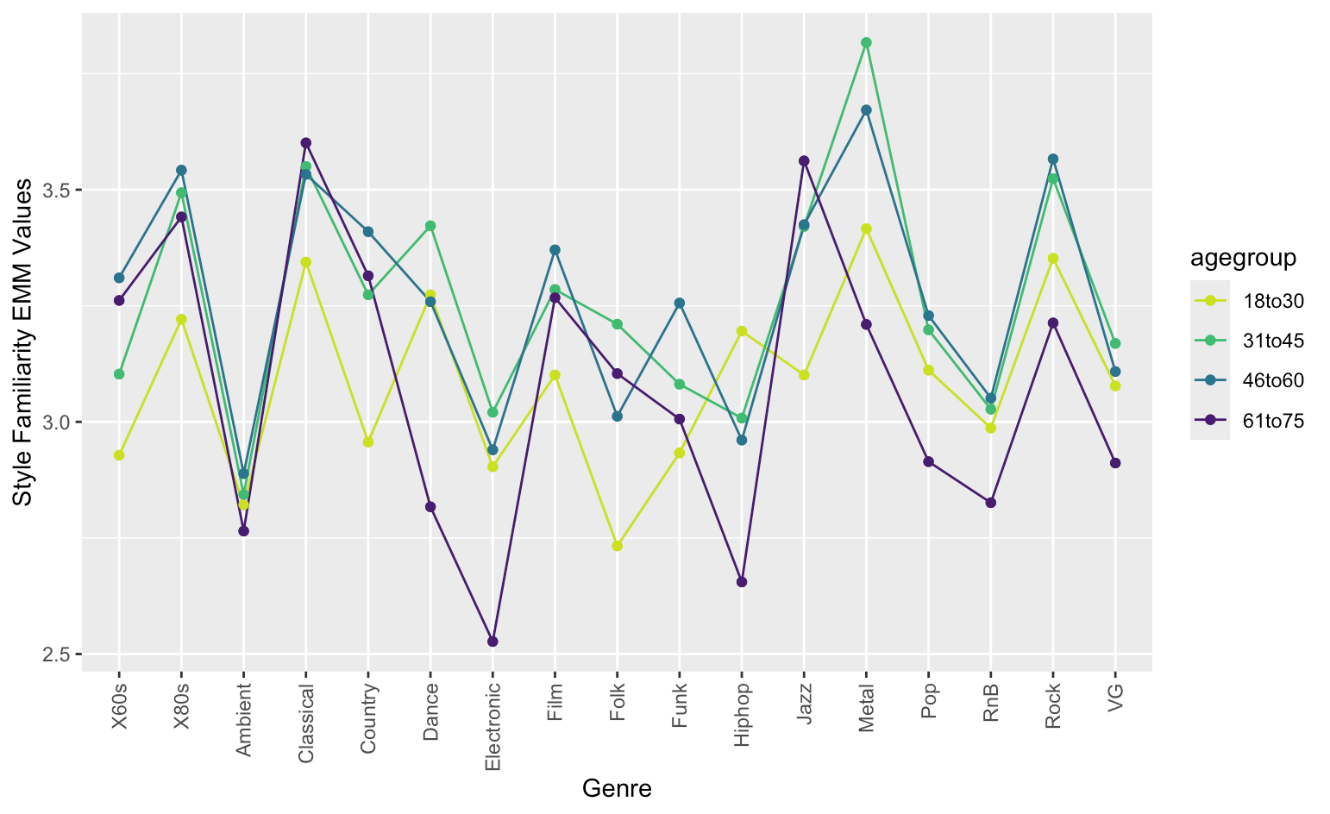


*Supplementary Figure 4. ANOVA plot for the effect of nationality on style familiarity ratings across genres*
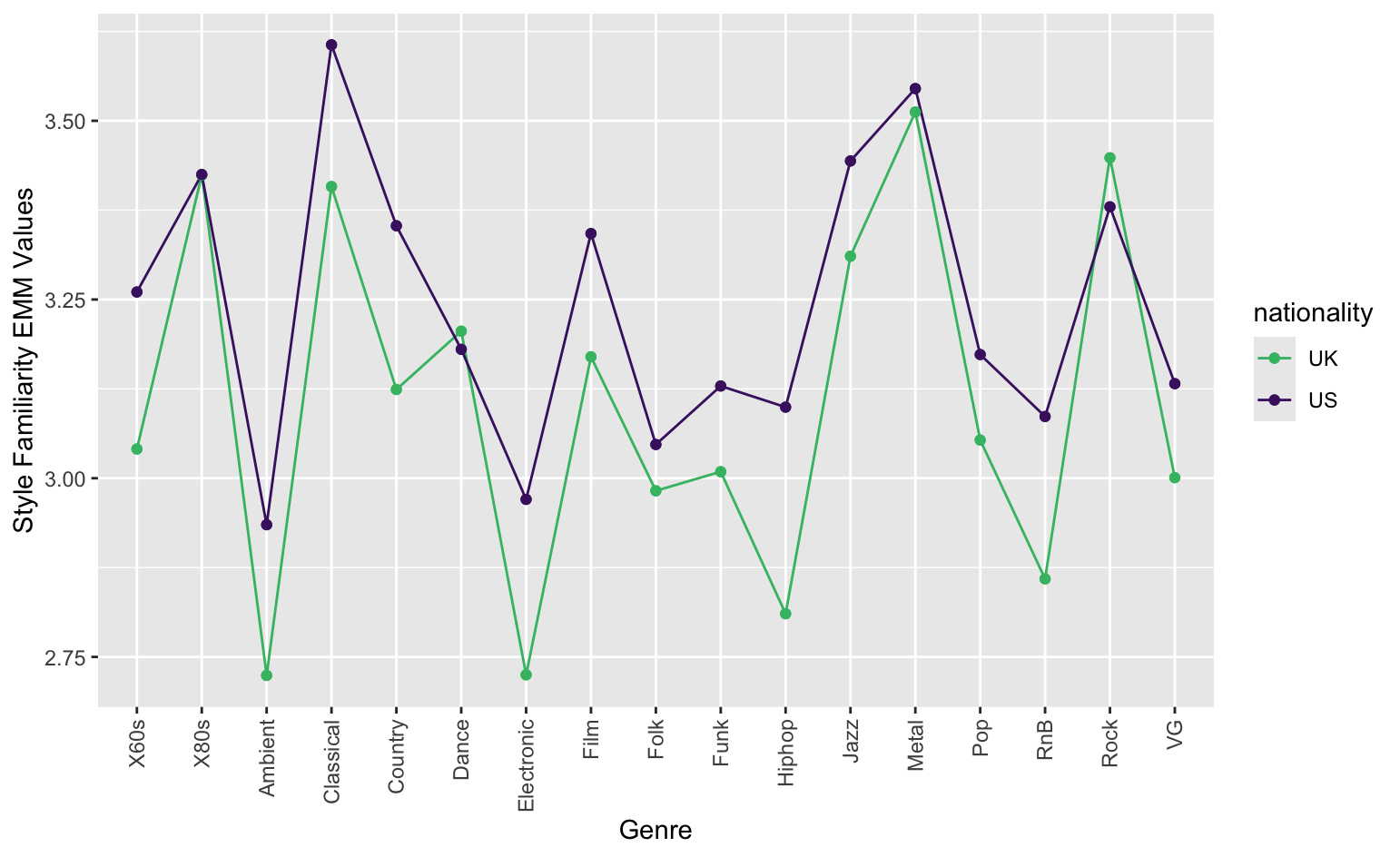


*Supplementary Figure 5. ANOVA plot for the effect of age group on enjoyment ratings across genres*


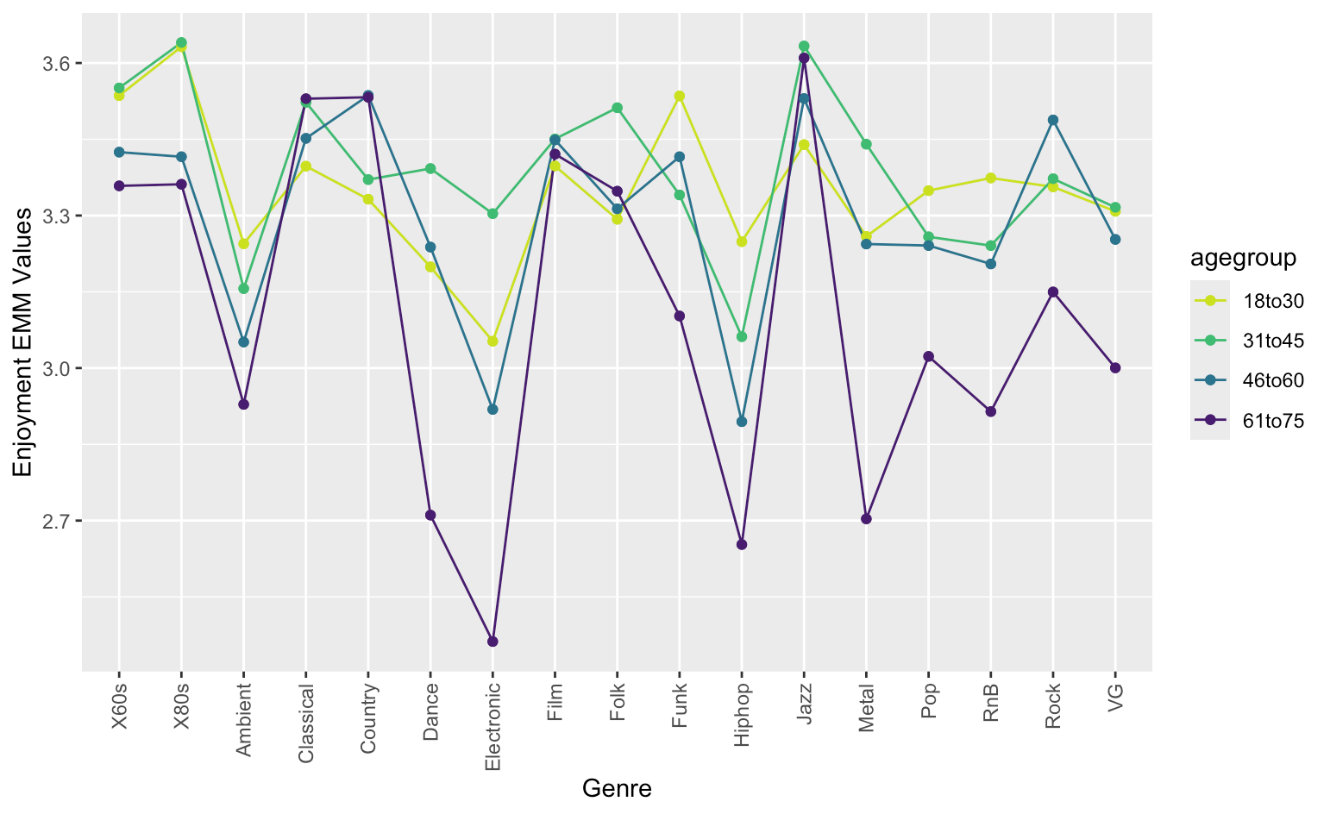
*Supplementary Figure 6. ANOVA plot for the effect of nationality on enjoyment ratings across genre*


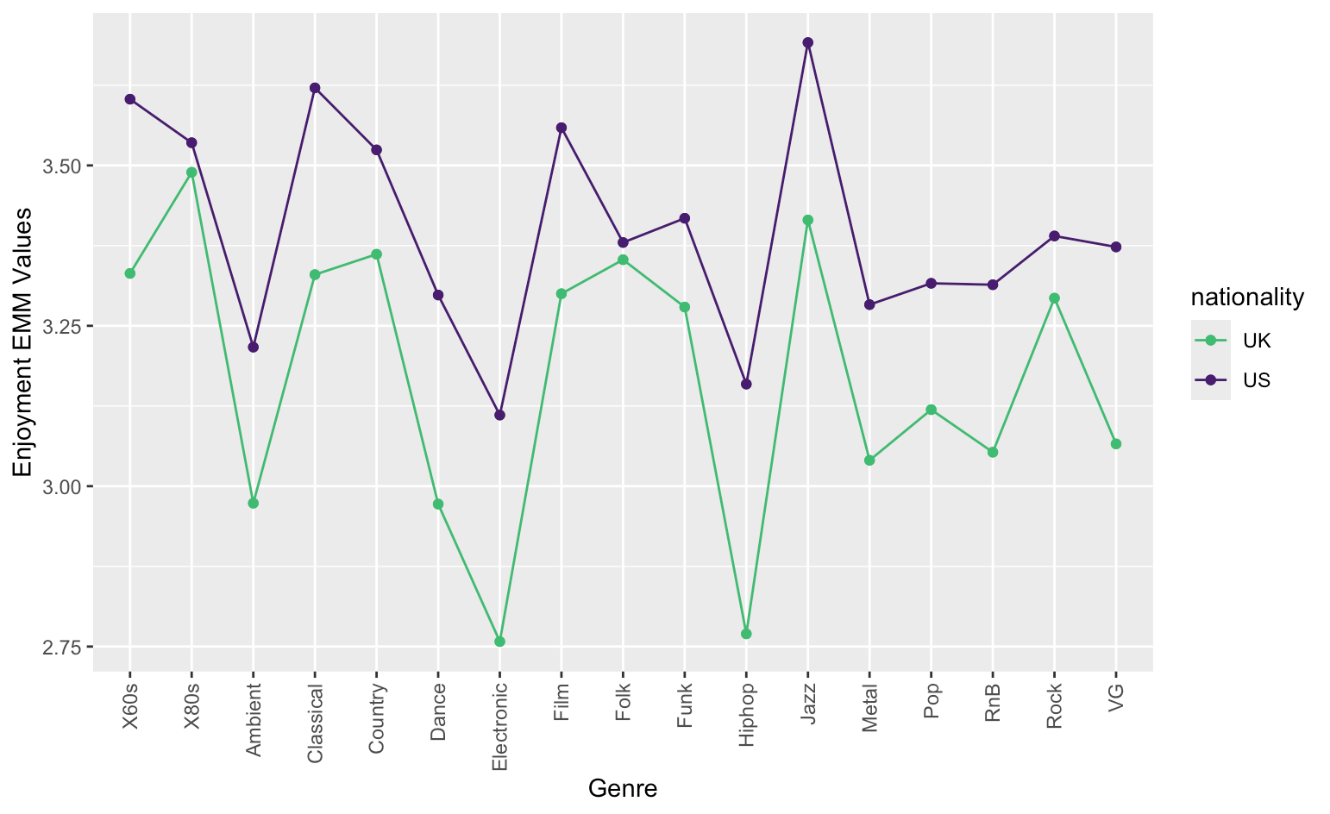


*Supplementary Figure 7. ANOVA plot for the effect of age group on valence ratings across genre*


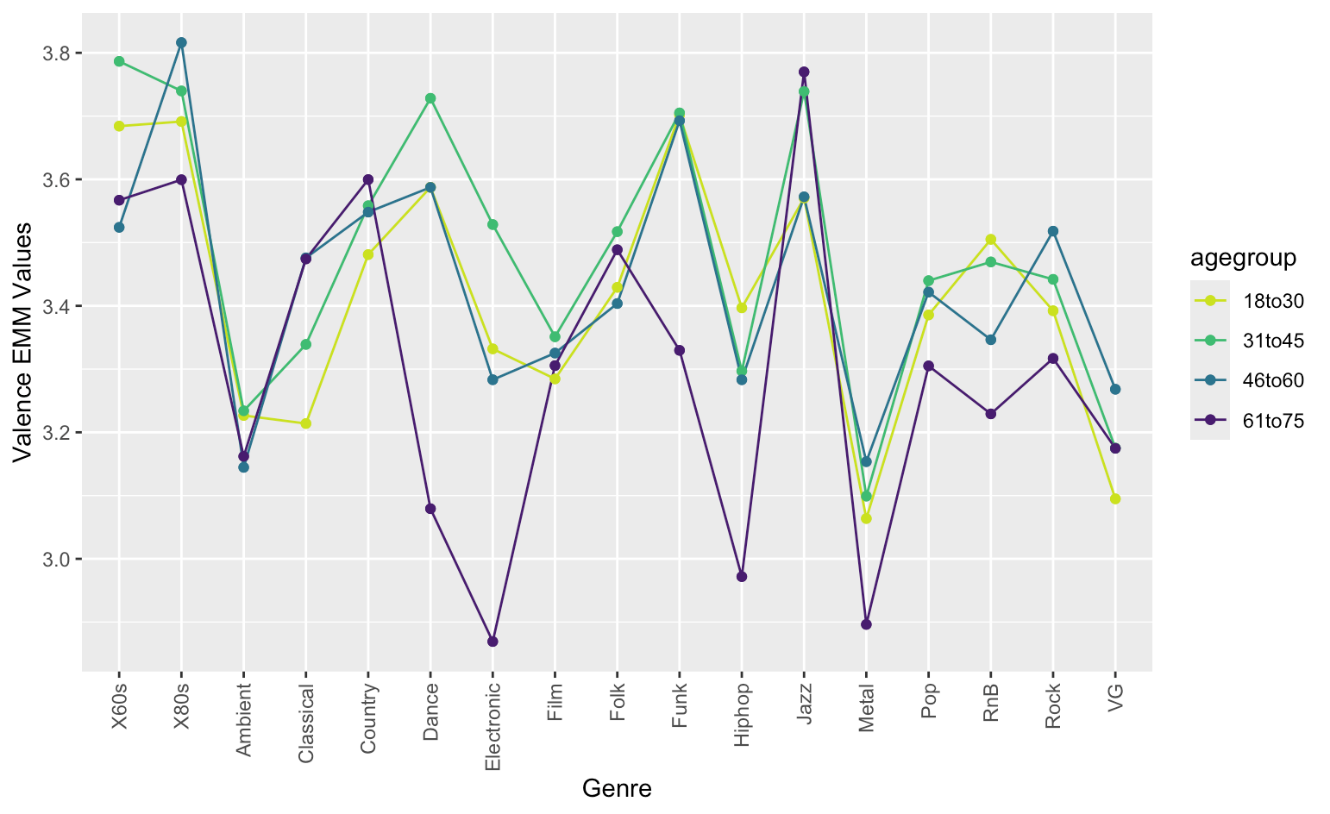


*Supplementary Figure 8. ANOVA plot for the effect of nationality on valence ratings across genres*
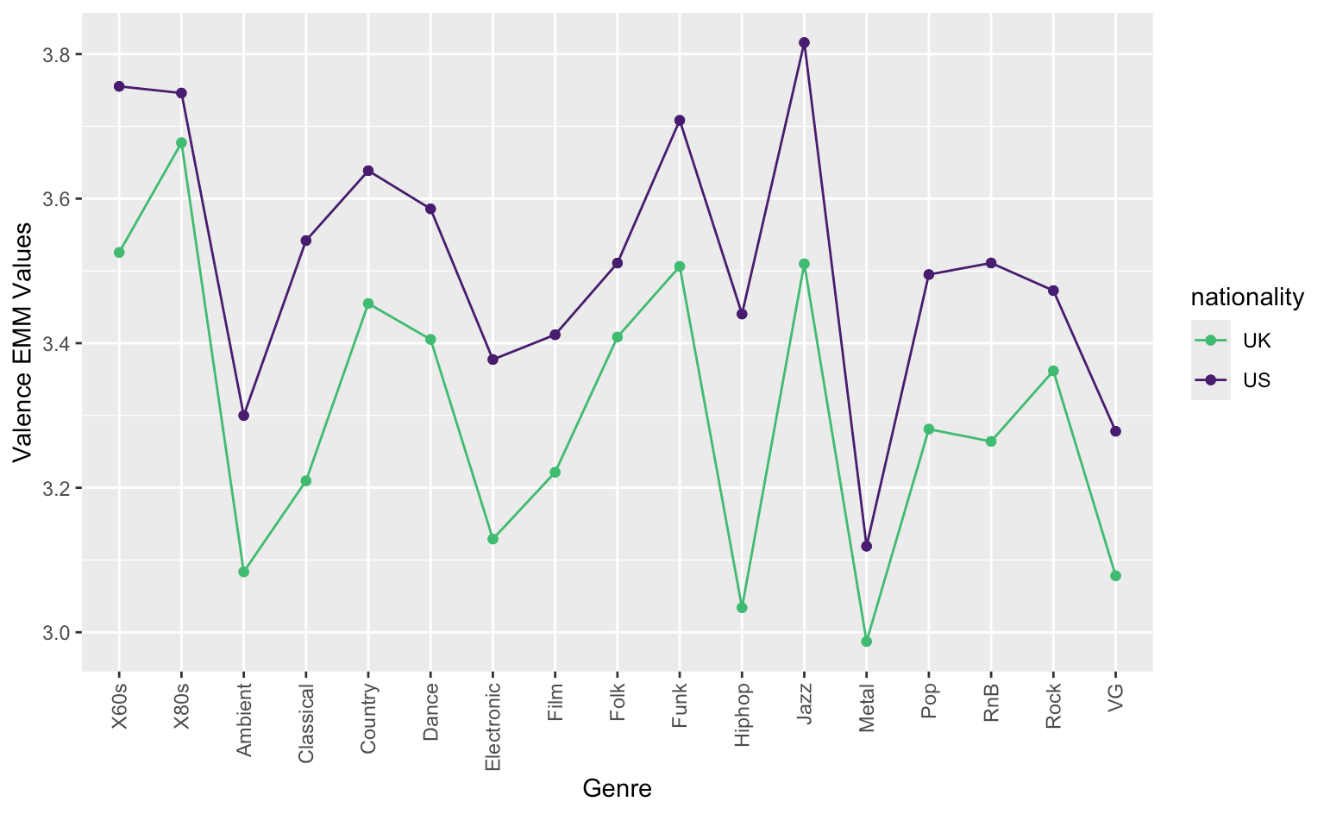


*Supplementary Figure 9. ANOVA plot for the effect of nationality on arousal ratings across genres*
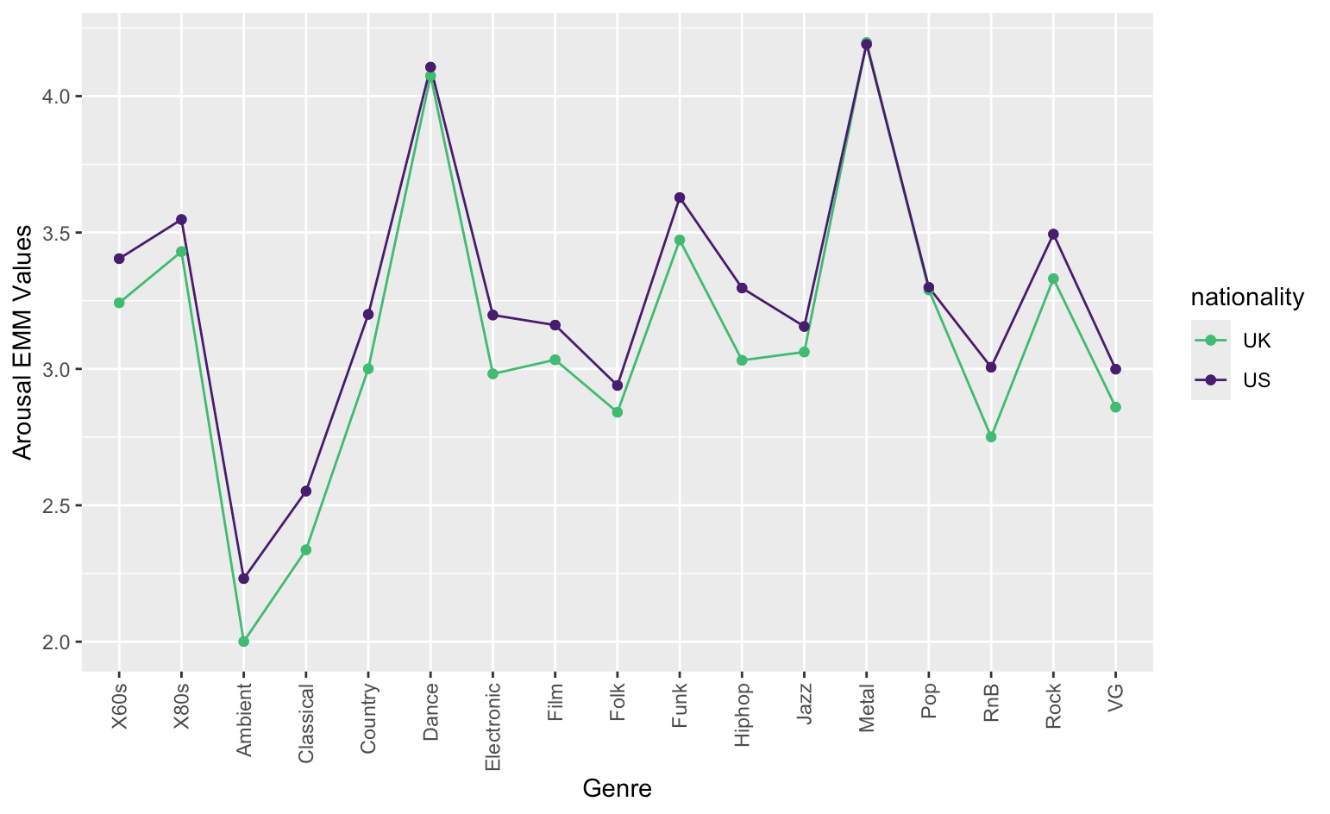

Supplement: Supplementary file 1 — Supplementary file1 (DOCX 3948 KB) [file 13428_2025_2724_MOESM1_ESM.docx]
